# A green and efficient synthetic methodology towards the synthesis of 1-allyl-6-chloro-4-oxo-1,4-dihydroquinoline-3-carboxamide derivatives

**DOI:** 10.1186/s13065-022-00902-1

**Published:** 2022-12-08

**Authors:** Muhammad Shoaib Ali Gill, Nursyuhada Azzman, Sharifah Syed Hassan, Syed Adnan Ali Shah, Nafees Ahemad

**Affiliations:** 1 School of Pharmacy, Monash University Malaysia, Jalan Lagoon Selatan, Bandar Sunway, 47500 Petaling Jaya, Selangor DE Malaysia; 2 Jeffrey Cheah School of Medicine and Health Sciences, Monash University Malaysia, Jalan Lagoon Selatan, Bandar Sunway, 47500 Petaling Jaya, Selangor DE Malaysia; 3 Institute of Pharmaceutical Sciences, University of Veterinary and Animal Sciences, Syed Abdul Qadir Jillani, Out Fall Road, Lahore, Pakistan; 4 Faculty of Pharmacy, Universiti Teknologi MARA, Cawangan Pulau Pinang Kampus Bertam, 13200 Kepala Batas, Pulau Pinang Malaysia; 5 Faculty of Pharmacy, Universiti Teknologi MARA Cawangan Selangor Kampus Puncak Alam, 42300 Bandar Puncak Alam, Selangor DE Malaysia; 6 Tropical Medicine and Biology Multidisciplinary Platform, Monash University Malaysia, Jalan Lagoon Selatan, Bandar Sunway, 47500 Petaling Jaya, Selangor DE Malaysia

**Keywords:** Green synthesis, 4-Quinolone, N-Alkylation, Carboxamide, Scalable

## Abstract

Quinolone is a privileged scaffold in medicinal chemistry and 4-Quinolone-3-Carboxamides have been reported to harbor vast therapeutic potential. However, conversion of N-1 substituted 4-Quinolone 3-Carboxylate to its corresponding carbamates is highly restrictive. This motivated us to adopt a much simpler, scalable and efficient methodology for the synthesis of highly pure N-1 substituted 4- Quinolone-3-Carboxamides with excellent yields. Our adopted methodology not only provides a robust pathway for the convenient synthesis of N-1 substituted 4- Quinolone-3-Carboxamides which can then be explored for their therapeutic potential, this may also be adaptable for the derivatization of other such less reactive carboxylate species.

**Supplementary Information:**

The online version contains supplementary material available at 10.1186/s13065-022-00902-1.

## Introduction

The 4-quinolone scaffold holds significant relevance in medicinal chemistry e.g. Flouro-quinolones are among the most important fully synthetic antibiotics. The quinolone itself is a privileged scaffold in terms of its druggability, finding its utility in drugs ranging from anticancer, antibacterial and antiviral to cystic fibrosis and cardiotonic. This importance also highlighted by the fact that Quinolone and its allied scaffolds are found amongst more than 60 FDA approved drugs [1, 2]. Furthermore 4-quinolones, in appreciable number have been obtained from biological sources and reported for their antibacterial, antiplasmodial, and cytotoxic potentials. These isolated 4-quinolones are categorized by an alkyl or alkenyl group at C-2, and C-3, they serve as lead structures for synthetic anti-microbial agents, some of them with very novel mechanisms of action such as quorum sensing signaling molecules controlling the population density of Pseudomonas *spp.* [3].

Many specific synthetic methodologies have been developed and reported for the production of quinolone antibiotics [4, 5]. These methods range from multi-stepped, one-pot, flow chemistry and metal catalyzed reactions resulting in targeted modification at C2, C3 or N-hydroxylation [6–9]. In terms of C3 substitution on the 4-quinolone nucleus, mostly C3 carboxylic acid derivatives have been explored with modification ranging from N1 to C8 for anti-biotic and antivirals [5, 10–12].

**Scheme 1:**
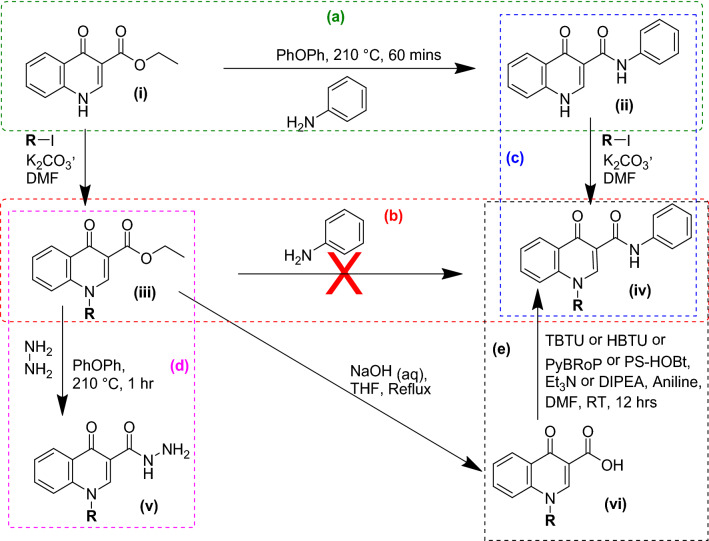
Reported methods for the synthesis of Carboxamides **(ii** & **iv)** from ethyl 4-oxo-1,4-dihydroquinoline-3-carboxylate **(i)** and N-1 substituted 4-oxo-1,4-dihydroquinoline-3-carboxylate **(iii)** respectively

4-Quinolone-3-carboxamides have been explored for their anti-tubercular [13], anti-proliferative [14, 15], tyrosine kinase inhibition [16], and anti-inflammatory potential (via Cannabinoid receptor 2 ligand) [17, 18]. In-silico studies have also identified promising anti-cancer leads with 4-Quinolone-3-carboxamides scaffold [19]. The conversion of 4-Quinolone-3-carboxylate **(i)** into corresponding 4-Quinolone-3-carboxamides **(ii)** has been achieved by direct thermal coupling of various amines (Scheme 1a). Various such carboxamide derivatives have been reported and tested for their anti-neoplastic potential [20]. However, in case of N-1 substituted 4-oxo-1,4-dihydroquinoline-3-carboxylate **(iii)**, the substitution at N-1 leads to a loss of acidity, resulting in a loss of reactivity at the 3-Carboxylate end and hence the direct coupling with an amine to produce the resultant N-1 substituted 4-oxo-1,4-dihydroquinoline-3-carboxamide **(iv)** is not possible (Scheme 1b). One way to overcome this loss of reactivity is that the N-1 substitution can be done after 3-Carboxamide **(iii)** moiety is synthesized (Scheme 1c) [21–24]. However, this is sometimes not viable as the carbamate nitrogen in **(ii)** may also present itself as a competing target rather than the intended N-1. However N-1 substituted 4-oxo-1,4-dihydroquinoline-3-carbohydrazide **(v)** were an exception to this (Scheme 1d) [25]. Alternatively, N-1 substituted 4-oxo-1,4-dihydroquinoline-3-carboxamide **(iv)** can also be produced by the use of a peptide coupling agent such as TBTU, HBTU or PyBRoP, PS-HOBt under alkaline conditions (Scheme 1e) albeit only after converting the N-substituted 4-Quinolone-3-carboxylate **(iii)** into the corresponding carboxylic acid **(vi)** [16, 26–29].

These synthetic methods although viable are multistep, and costly as they require specialized coupling agents which can be costly. Moreover, they sometimes require elaborate isolation techniques such as column chromatography; this makes not only the task laborious but more importantly leads to reduced yields of the final product. Here in we report the exploration and optimization of an adapted synthetic methodology for the synthesis of 1-allyl-6-chloro-4-oxo-N-phenyl-1,4-dihydroquinoline-3-carboxamides with excellent yields and high purity, using a wide range of anilines and benzyl amines.

## Results and discussion

**Scheme 2:**
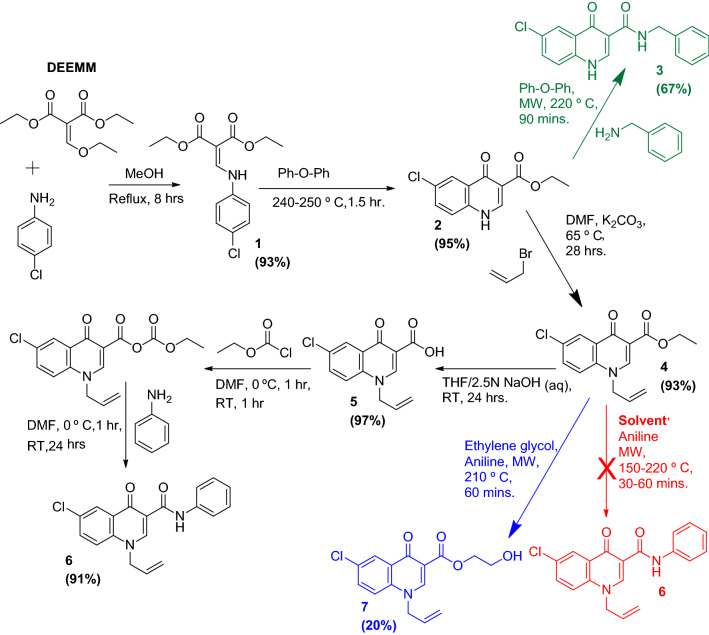
Optimized synthesis of 1-allyl-6-chloro-4-oxo-1,4-dihydroquinoline-3-carboxamide derivatives

We begin with the synthesis of anilinomethylenemalonate **1**, by refluxing diethyl ethoxymethylenemelonate (DEEMM, 1.05 equivalent) with 4-chloroaniline (Scheme 2). Both microwave assisted synthesis (Anton Paar Monowave 400) and conventional heating were employed and the later method was inferred to be more efficient (Table 1). The enclosed vessel used for the microwave reaction did not allow for the Ethanol (EtOH) by-product to escape thus not limiting the back reaction, however the open vessel used for conventional heating did allow the escape of high energy EtOH molecules thus facilitating the forward reaction. The reaction was monitored through TLC, methanol proved to be the best solvent most probably because of the lack of solubility of the product in it. To get the best yields with alcoholic solvents, the reaction mixture was brought to room temperature, quenched with cold water, filtered and dried.

**Table 1:** Reaction conditions for the synthesis of Anilinomethylenemalonate **(1)**

Entry	Solvent	Heating method	Temperature/Time	% Yield
1	MeOH	Microwave	120 ℃ for 30 min	56
2	MeOH	Conventional	Reflux, 4 h	78
3	MeOH	Conventional	Reflux, 8 h	93
4	EtOH	Microwave	140 ℃ for 30 min	32
5	EtOH	Conventional	Reflux, 4 h	63
6	EtOH	Conventional	Reflux, 8 h	84
7	DCM	Conventional	Reflux, 4 h	20
8	DCM	Conventional	8 h	32

Gould-Jacobs reaction was employed for synthesis of ethyl 6-chloro-4-oxo-1,4-dihydroquinoline-3-carboxylate **(2)** (Scheme 2). Again both Conventional heating and microwave irradiation were used. In Anton Paar Monowave 400 microwave reactor G30 vial 2 gm of **1** was mixed in 10 mL of diphenyl ether (PhOPh) and irradiated to 250 ℃ for 1 h. A dark precipitous solution formed within the vial on cooling. 10 mL of ethyl acetate was added and stirred for an hour. The product **2** was filtered under vacuum, washed with ethyl acetate and dried to obtain product in 53% yield.

Conventional thermal cyclization of enamine to yield the 4-Quinolone **(2)** was also carried out in an open conical flask or a beaker. The Anilinomethylenemalonate (**1)** was suspended in diphenyl ether (ratio; 2gm/10 mL) and the mixture was stirred and heated to 240–250 ℃ for 1.5 h. The dark mixture was cooled to room temperature, diluted with ample amount of ethyl acetate and stirred overnight. The residue was filtered and dried in air. To remove the residual diphenyl ether, the residue was re-suspended in boiling ethyl acetate cooled to ambient temperature, filtered under vacuum and dried to yield pure **2** in near quantitative yield 95%. Conventional method yielded better results and hence it was adopted for all the later such reactions.

The Quinolones carboxylate **(2)** was coupled with benzyl amine with slight modification to the reported procedure [20]. Anton Paar Monowave 400 microwave reactor was employed at 220 ℃ for 1.5 h. The close reaction vial allowed for the reaction to be performed with milli-molar quantities, providing good yields and employing lesser amount of solvent (PhOPh, 4 mL). After bringing the reaction mixture to room temperature 6 mL of EtOH was added to the vial and stirred overnight. Precipitate was filtered dried and recrystallized with EtOH to give pure N-benzyl-6-chloro-4-oxo-1,4-dihydroquinoline-3-carboxamide **(3)**, yield 67%. This served as a baseline to establish the C3 reactivity of N-1 unsubstituted 4-Quinolone nucleus.

N-allyl substitution of **2** was done by mixing **2** (11.9 mmol, 3 gm) and anhydrous K_2_CO_3_ (18 mmol, 2.5 gm) in a round bottom flask; dry *N,N*-Dimethylformamide (DMF) 50 mL was employed as solvent. Allyl bromide (14.3 mmol, 1.24 mL) was added dropwise while stirring. Catalytic amount of NaI was added and the reaction was than heated in a reflux at 65 ℃ for 28 h. The reaction was monitored through TLC and upon completion, brought to room temperature and quenched with ice cold water (500 mL). The precipitate was filtered, dried and recrystallized to give ethyl 1-allyl-6-chloro-4-oxo-1,4-dihydroquinoline-3-carboxylate **(4)** as white solid. Yield: 93%. m.p. 170–172 ℃.

To perform saponification of **4** (3.83 mmol, 1.19 gm), tetrahydrofuran (THF) 10 mL was added and stirred in a flask for 10 min. Later, 10 mL of 2.5 N NaOH aqueous solution was added to the above mixture and stirred at room temperature for 24 h. The reaction progression monitored via TLC and upon completion THF was removed under vacuum and the solution was titrated to pH 4–5 using 5 N HCl solution. The precipitate was filtered, washed capaciously with water and dried to give 1-allyl-6-chloro-4-oxo-1,4-dihydroquinoline-3-carboxylic acid **(5)** as pinkish solid. Yield: 97%. m.p. 233–235 ℃.

Finally, **5 (**1.2 mmol, 0.32 gm) was dissolved in 10 mL of anhydrous DMF in a round bottom flask. Triethylamine (3 mmol, 426 µL) was added, the mixture was cooled to 0 ℃ and then stirred for 30 min. Ethylchloroformate (2.4 mmol, 231 µL) was added dropwise and stirred for 1 h at 0 ℃ and another hour at room temperature. The reaction mixture was again brought to 0 ℃, before the addition of amine (Aniline, 2.4 mmol, 222 µL) dropwise, the reaction was stirred at 0 ℃ for an hour before being brought to room temperature and stirred for 24 h. The reaction progression was monitored via TLC. Once complete the reaction was quenched by pouring into 100 mL ice cold aqueous 0.5 N NaOH solution and stirred vigorously overnight. The precipitate was filtered and dried to yield 1-allyl-6-chloro-4-oxo-N-phenyl-1,4-dihydroquinoline-3-carboxamide **(6)**. The product was recrystallized with ethanol. The use of 0.5 N NaOH allowed for the removal of any unreacted Quinolone Carboxylic acid **5** or the amine carbamate which could have formed as a byproduct due to the excess use of ethylchloroformate and amine. The result was a highly pure product with excellent overall yields.

Initially direct coupling of **4** with aniline was also attempted to achieve **6** via microwave assisted irradiation. However, despite using different solvents and reaction conditions, direct synthesis was not achieved (Table 2). Interestingly, when ethylene glycol was used as solvent the final product isolated was an ester, 2-hydroxyethyl 1-allyl-6-chloro-4-oxo-1,4-dihydroquinoline-3-carboxylate **(7)**, which can be explored further for chemical and biological potential.

**Table 2:** Attempted microwave assisted coupling of ethyl 1-allyl-6-chloro-4-oxo-1,4-dihydroquinoline-3-carboxylate **(4)** with aniline

Entry	Solvent	Temperature (℃)	Time (minutes)	Reaction
1	EtOH	150	30	No-reaction
2	EtOH	150	60	No-reaction
3	Ethylene glycol	200	30	Compound 7 isolated
4	DMSO	180	30	No-reaction
5	DMSO	200	30	•Slight reaction on TLC •Mostly unreacted •Not isolatable •No appreciable change with change in reaction conditions
6	DMSO	200	60	
7	DMF	200	30	
8	DMF	200	60	
9	DMF	220	60	
10	PhOPh	220	30	No reaction
11	PhOPh	220	60	No reaction

**Fig. 1:**
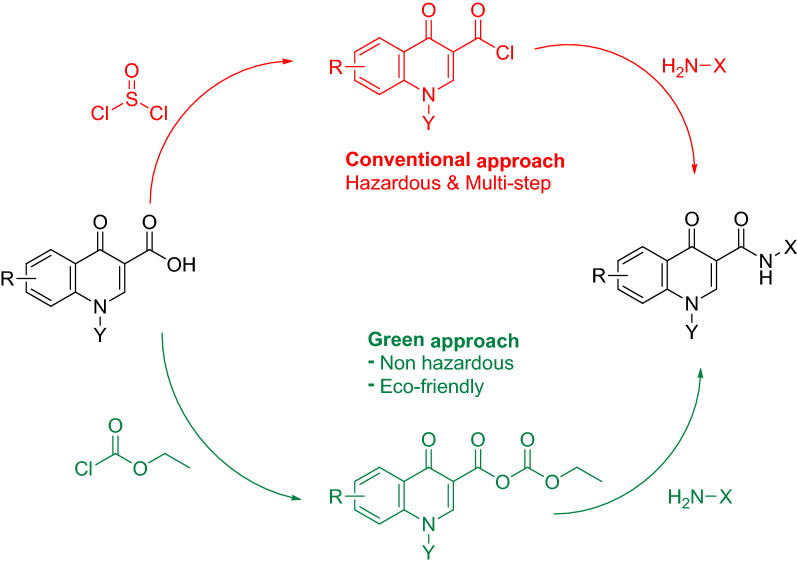
Alternative synthesis approaches for 4-Hydroxy Quinolones Carboxamides

One possible explanation for the failure of direct coupling between the aniline and **4** is; when Quinolone ring Nitrogen was allylated this led to a loss of acidity and resultantly a loss of reactivity at the C-3 carboxylate end. This prompted the need for an alternative path [30] to be adopted for the synthesis of respective carboxamides (Scheme 2). The N-substituted Quinolone carboxylate was 1st converted into the corresponding carboxylic acid. Conventionally, the acid can be converted to acid chloride and then reacted with amine to yield the Carboxamide. This method while viable; is multistep, drastic and environmentally non-friendly. We thus, opted for a greener approach by converting the N-substituted quinolone carboxylic acid into corresponding anhydride by reacting it with acyl chloride and lastly introducing the amine into the reaction mixture (Fig. 1).

**Scheme 3:**
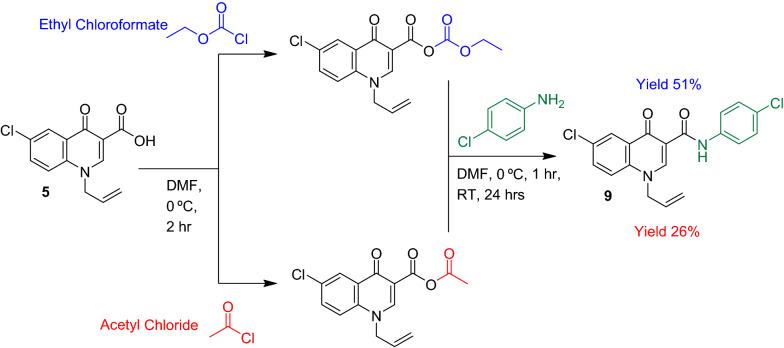
Comparison of acylating agents Ethylchloroformate and Acetyl chloride

Moreover, two different acylation agents Acetyl chloride and Ethylchloroformate were used (Scheme 3). Ethylchloroformate was found to be more effective this was doubly advantageous as Acetyl chloride is a controlled substance and requires specialized import permission and is transported only via sea freight.

The Ethylchloroformate mediated synthesis of 4-hydroxy quinolones carboxamides as shown in Scheme 2 was optimized for the ratio of ethylchloroformate, aniline and triethylamine as shown in Table 3.

**Table 3:** Optimization of adapted synthesis as depicted in Scheme 2

Entry	Solvent	Triethylamine ratio	Ethylchloroformate ratio	4-Cl-aniline Ratio	% Yield
1	DMF	1.5	1.2	1.2	51
2	DMF	2	1.5	1.5	78
3	DMF	2.5	2	2	90
4	DMSO	2.5	2	2	87

Note: DMF was selected for further synthesis due to the ease of final aqueous extraction

**Fig. 2:**
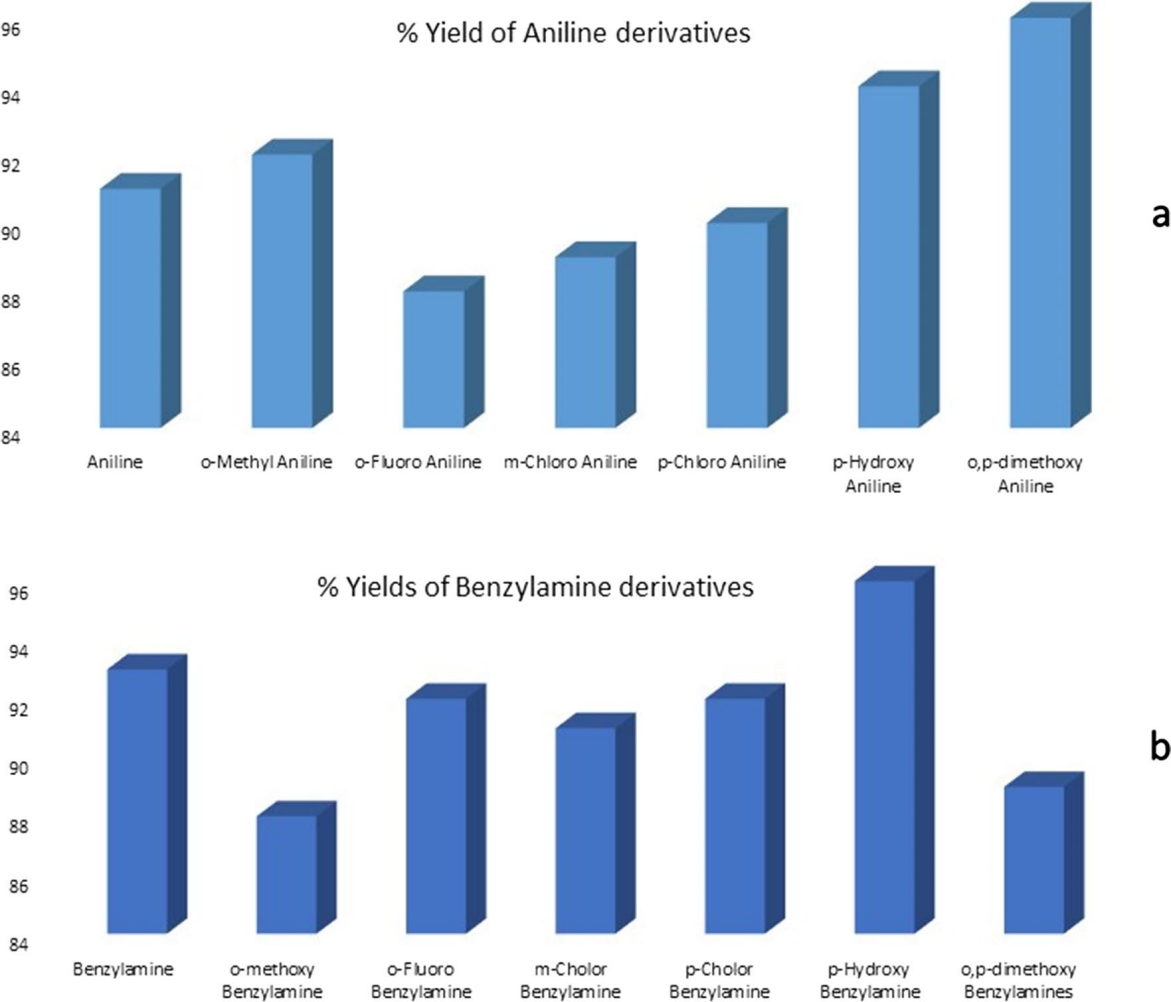
Reactivity trend for various substituted **a** Anilines and **b** Benzyl amines when reacted in accordance with Scheme-1

To explore the validity of the synthesis methodology, different substituted anilines and corresponding benzyl amines were reacted with **5** leading to the synthesis of derivatives **8–20** (Table 4). The synthesis of all **8–20** was carried out as per the procedure adopted for synthesis of **6** in Scheme 2. In terms of reactivity unsubstituted and para substituted anilines provided high yields (Fig. 2a), whereas for benzyl amines the *m*-substituted benzyl amines fared far better when compared with the corresponding *m-*anilines (Fig. 2b). The fair to excellent yields of the synthesized carboxamide derivatives also emphasizes the robustness of this adopted methodology.

**Table 4:** Synthesized carboxamide derivatives (**8–20**) of 1-allyl-6-chloro-4-oxo-1,4-dihydroquinoline-3-carboxylic acid (**5)**


Compound ID (% Yield)	Substituent X	Compound ID (% Yield)	Substituent X	Compound ID (% Yield)	Substituent X
**8 (**93%**)**		**9 (**90%**)**		**10 (**90%**)**	
**11 (**89%**)**		**12 (**91%**)**		**13 (**82%**)**	
**14 (**90%**)**		**15 (**96%**)**		**16 (**89%**)**	
**17 (**94%**)**		**18 (**96%**)**		**19 (**88%**)**	
		**20 (**92%**)**

## Conclusions

The N-substituted 4-Quinolone-3-Carboxylate tends to have restricted reaction potential at the C-3 carboxylate requiring specialized reagents and sometimes drastic and complex reaction and extraction methodology. We have explored and tuned an adapted methodology for the synthesis of N-1 substituted 4-Quinolone-3-Carboxamides. The reaction proves to be highly efficient and robust with inherent mechanisms to ensure the quality of the product, also it is reproducible when explored for wide range of functionalized anilines and benzyl amines. The near quantitative yields and the high purity achieved through this methodology shows great potential in organic synthesis. This can pave the way for the convenient synthesis of a whole range of therapeutically interesting small molecules with privileged scaffold such as 4-Quinolone or other carboxylates by extension.

## Supplementary Information

**Additional file 1: Figure S1.** ^1^H-NMR Spectra of 1. **Figure S2.** ^1^H-NMR Spectra of 2. **Figure S3.** ^1^H-NMR Spectra of 3. **Figure S4.** ^1^H-NMR Spectra of 4. **Figure S5.** ^1^H-NMR Spectra of 5. **Figure S6:** ^1^H-NMR Spectra of 6. **Figure S7:** ^1^H-NMR Spectra of 7. **Figure S8.** ^1^H-NMR Spectra of 8. **Figure S9:** ^1^H-NMR Spectra of 9. **Figure S10.** ^1^H-NMR Spectra of 10. **Figure S11:** ^1^H-NMR Spectra of 11. **Figure S12.** ^1^H-NMR Spectra of 12. **Figure S13.** ^1^H-NMR Spectra of 13. **Figure S14.** ^1^H-NMR Spectra of 14. **Figure S15:** ^1^H-NMR Spectra of 15. **Figure S16:** ^1^H-NMR Spectra of 16. **Figure S17.** ^1^H-NMR Spectra of 17. **Figure S18.** ^1^H-NMR Spectra of 18. **Figure S19.** ^1^H-NMR Spectra of 19. **Figure S20.** ^1^H-NMR Spectra of 20. **Figure S21a.** LCMS Data of 3. **Figure S21b.** MS Data plot of 3. **Figure S22.** HPLC Data of 4. **Figure S23.** HPLC Data of 5. **Figure S24.** HPLC Data of 6. **Figure S25a.** LCMS Data of 7. **Figure S25b.**MS Data plot of 7. **Figure S26.** HPLC Data of 8. **Figure S27.** HPLC Data of 9. **Figure S28.** HPLC Data of 10. **Figure S29.** HPLC Data of 11. **Figure S30.** HPLC Data of 12. **Figure S31.** HPLC Data of 13. **Figure S32.** HPLC Data of 14. **Figure S33.** HPLC Data of 15. **Figure S34.** HPLC Data of 16. **Figure S35.** HPLC Data of 17. **Figure S36.** HPLC Data of 18. **Figure S37.** HPLC Data of 19. **Figure S38.** HPLC Data of 20.

## Data Availability

Supplementary data to this article can be found as Additional file 1 online. The datasets generated and/or analyzed during the current study are not publicly available but are available from the corresponding author on reasonable request.
