# Supplementary material for: A green and efficient synthetic methodology towards the synthesis of 1-allyl-6-chloro-4-oxo-1,4-dihydroquinoline-3-carboxamide derivatives
Source: BMC Chem. 2022 Dec 8;16(1):111. doi: 10.1186/s13065-022-00902-1 (PMC9733071; doi:10.1186/s13065-022-00902-1)
Supplement: Supplementary file 1 — Additional file 1: Figure S1. 1H-NMR Spectra of 1. Figure S2. 1H-NMR Spectra of 2. Figure S3. 1H-NMR Spectra of 3. Figure S4. 1H-NMR Spectra of 4. Figure S5. 1H-NMR Spectra of 5. Figure S6: 1H-NMR Spectra of 6. Figure S7: 1H-NMR Spectra of 7. Figure S8. 1H-NMR Spectra of 8. Figure S9: 1H-NMR Spectra of 9. Figure S10. 1H-NMR Spectra of 10. Figure S11: 1H-NMR Spectra of 11. Figure S12. 1H-NMR Spectra of 12. Figure S13. 1H-NMR Spectra of 13. Figure S14. 1H-NMR Spectra of 14. Figure S15: 1H-NMR Spectra of 15. Figure S16: 1H-NMR Spectra of 16. Figure S17. 1H-NMR Spectra of 17. Figure S18. 1H-NMR Spectra of 18. Figure S19. 1H-NMR Spectra of 19. Figure S20. 1H-NMR Spectra of 20. Figure S21a. LCMS Data of 3. Figure S21b. MS Data plot of 3. Figure S22. HPLC Data of 4. Figure S23. HPLC Data of 5. Figure S24. HPLC Data of 6. Figure S25a. LCMS Data of 7. Figure S25b.MS Data plot of 7. Figure S26. HPLC Data of 8. Figure S27. HPLC Data of 9. Figure S28. HPLC Data of 10. Figure S29. HPLC Data of 11. Figure S30. HPLC Data of 12. Figure S31. HPLC Data of 13. Figure S32. HPLC Data of 14. Figure S33. HPLC Data of 15. Figure S34. HPLC Data of 16. Figure S35. HPLC Data of 17. Figure S36. HPLC Data of 18. Figure S37. HPLC Data of 19. Figure S38. HPLC Data of 20. [file 13065_2022_902_MOESM1_ESM.docx]

**Additional File 1
Supporting Information for:**

**A green and efficient synthetic methodology towards the synthesis of 1-allyl-6-chloro-4-oxo-1,4-dihydroquinoline-3-carboxamide derivatives**

Muhammad Shoaib Ali Gill^1,3^, Nursyuhada Azzman^1,4^, Sharifah Syed Hassan^2^, Syed Adnan Ali Shah^5^, Nafees Ahemad*^1,6^

^1^ School of Pharmacy, Monash University Malaysia, Jalan lagoon selatan, Bandar Sunway, Petaling Jaya 47500, Selangor DE, Malaysia.

^2^ Jeffrey Cheah School of Medicine and Health Sciences, Monash University Malaysia, Jalan lagoon selatan, Bandar Sunway, Petaling Jaya 47500, Selangor DE, Malaysia.

^3^ Institute of Pharmaceutical Sciences, University of Veterinary and Animal Sciences, Syed Abdul Qadir Jillani (Out Fall) Road, Lahore, Pakistan.

^4^ Faculty of Pharmacy, Universiti Teknologi MARA, Cawangan Pulau Pinang Kampus Bertam, 13200 Kepala Batas, Pulau Pinang, Malaysia.

^5^ Faculty of Pharmacy, Universiti Teknologi MARA Cawangan Selangor Kampus Puncak Alam, Bandar Puncak Alam 42300, Selangor DE, Malaysia.

^6^ Tropical Medicine and Biology Multidisciplinary platform, Monash University Malaysia, Jalan lagoon selatan, Bandar Sunway, Petaling Jaya 47500, Selangor DE, Malaysia.

Contents

[1 General Experimental Material and Method 5](#_Toc116215785)

[2 Synthesis methodology for the synthesis of 1-allyl-6-chloro-4-oxo-1,4-dihydroquinoline-3-Carboxamides 5](#_Toc116215786)

[2.1 Procedure for the synthesis of *diethyl 2-(((4-chlorophenyl) amino) methylene) malonate (1)* 5](#_Toc116215787)

[2.2 Procedure for the synthesis *ethyl 6-chloro-4-oxo-1,4-dihydroquinoline-3-carboxylate (2)* 5](#_Toc116215788)

[2.3 Procedure for the synthesis of *N-benzyl-6-chloro-4-oxo-1,4-dihydroquinoline-3-carboxamide (3)* 6](#_Toc116215789)

[2.4 Synthesis of *ethyl 1-allyl-6-chloro-4-oxo-1,4-dihydroquinoline-3-carboxylate (4)* 6](#_Toc116215790)

[2.5 Synthesis of *1-allyl-6-chloro-4-oxo-1,4-dihydroquinoline-3-carboxylic acid (5)* 6](#_Toc116215791)

[2.6 Synthesis of *1-allyl-6-chloro-4-oxo-N-phenyl-1,4-dihydroquinoline-3-carboxamide (6)* 7](#_Toc116215792)

[2.7 Unintended synthesis of *2-hydroxyethyl 1-allyl-6-chloro-4-oxo-1,4-dihydroquinoline-3-carboxylate (7)* 7](#_Toc116215793)

[2.8 Synthesis of 1-allyl-6-chloro-4-oxo-1,4-dihydroquinoline-3-Carboxamides (8-20) 8](#_Toc116215794)

[3 ^1^H-NMR data for Compounds 1-20 8](#_Toc116215795)

[Figure S1: ^1^H-NMR Spectra of 1 17](#_Toc116215796)

[Figure S2: ^1^H-NMR Spectra of 2 18](#_Toc116215797)

[Figure S3: ^1^H-NMR Spectra of 3 19](#_Toc116215799)

[Figure S4: ^1^H-NMR Spectra of 4 20](#_Toc116215800)

[Figure S5: ^1^H-NMR Spectra of 5 21](#_Toc116215801)

[Figure S6: ^1^H-NMR Spectra of 6 22](#_Toc116215802)

[Figure S7: ^1^H-NMR Spectra of 7 23](#_Toc116215803)

[Figure S8: ^1^H-NMR Spectra of 8 24](#_Toc116215804)

[Figure S9: ^1^H-NMR Spectra of 9 25](#_Toc116215805)

[Figure S10: ^1^H-NMR Spectra of 10 26](#_Toc116215806)

[Figure S11: ^1^H-NMR Spectra of 11 27](#_Toc116215807)

[Figure S12: ^1^H-NMR Spectra of 12 28](#_Toc116215808)

[Figure S13: ^1^H-NMR Spectra of 13 29](#_Toc116215809)

[Figure S14: ^1^H-NMR Spectra of 14 30](#_Toc116215810)

[Figure S15: ^1^H-NMR Spectra of 15 31](#_Toc116215811)

[Figure S16: ^1^H-NMR Spectra of 16 32](#_Toc116215812)

[Figure S17: ^1^H-NMR Spectra of 17 33](#_Toc116215813)

[Figure S18: ^1^H-NMR Spectra of 18 34](#_Toc116215814)

[Figure S19: ^1^H-NMR Spectra of 19 35](#_Toc116215815)

[Figure S20: ^1^H-NMR Spectra of 20 36](#_Toc116215816)

[3.1 LCMS analysis methodology 37](#_Toc116215817)

[3.2 HPLC analysis methodology 37](#_Toc116215818)

[3.2.1 HPLC/LCMS Data Table 38](#_Toc116215819)

[Figure S21a: LCMS Data of 3 39](#_Toc116215820)

[Figure S21b: MS Data plot of 3 40](#_Toc116215821)

[Figure S22: HPLC Data of 4 41](#_Toc116215822)

[Figure S23: HPLC Data of 5 42](#_Toc116215823)

[Figure S24: HPLC Data of 6 43](#_Toc116215824)

[Figure S25a: LCMS Data of 7 44](#_Toc116215826)

[Figure S25b: MS Data plot of 7 45](#_Toc116215827)

[Figure S26: HPLC Data of 8 46](#_Toc116215828)

[Figure S27: HPLC Data of 9 47](#_Toc116215829)

[Figure S28: HPLC Data of 10 48](#_Toc116215830)

[Figure S29: HPLC Data of 11 49](#_Toc116215831)

[Figure S30: HPLC Data of 12 50](#_Toc116215832)

[Figure S31: HPLC Data of 13 51](#_Toc116215833)

[Figure S32: HPLC Data of 14 52](#_Toc116215834)

[Figure S33: HPLC Data of 15 53](#_Toc116215835)

[Figure S34: HPLC Data of 16 54](#_Toc116215836)

[Figure S35: HPLC Data of 17 55](#_Toc116215837)

[Figure S36: HPLC Data of 18 56](#_Toc116215838)

[Figure S37: HPLC Data of 19 57](#_Toc116215839)

[Figure S38: HPLC Data of 20 58](#_Toc116215840)

# General Experimental Material and Method

The ^1^H-NMR spectra were recorded on a Fourier 300 Bruker spectrophotometer (1H at 300 MHz) and Bruker DRX 500 MHz (1H at 500 MHz). The measuring temperature for all NMR spectrometers is 25 °C. All chemical shifts are given as δ value (ppm) with reference to tetramethylsilane (TMS) as an internal standard for CDCl_3_ and DMSO-D6.Stuart SMP40, melting point apparatus was used to determine melting points (uncorrected). Thin layer chromatography was performed on silica gel coated aluminum plates and viewed under UV light. Agilent Infinity Lab LC/MSD with Agilent 1260 Infinity II equipped with DAD WR detector, Acquity UPLC ethylene-bridged hybrid (BEH) C18 (2.1 × 50 mm2, 1.7 μm) column fitted with a C18 guard cartridge. Agilent HPLC 1200 fitted with Zorbax Eclips XDB-C18 Column (narrow bore 3.5-μm. 2.1 × 150 mm) was employed for HPLC analysis

# Synthesis methodology for the synthesis of 1-allyl-6-chloro-4-oxo-1,4-dihydroquinoline-3-Carboxamides

## Procedure for the synthesis of *diethyl 2-(((4-chlorophenyl) amino) methylene) malonate (1)*

*p*-Chloroaniline (52.3 mmol, 6.66 gm) was taken and dissolved in methanol 50 ml in a conical flask and Diethyl ethoxy-methylene malonate (DEEMM) (1.05 equiv. 11 ml) was added while stirring. The reaction was heated on a hotplate at 130 °C for 8 hours after which 20 ml MeOH was added. Once the reaction was cooled to room temperature it was refrigerated overnight. The crystals formed were crushed with a spatula, Ice cold water was added q.s. 100 ml and stirred rigorously for 10-20 minutes. The residue was filtered and washed with water and dried in vacuum with temperature not exceeding 50° C to give diethyl 2-(((4-chlorophenyl) amino) methylene) malonate (**1**) as white color solid. Yield: 93%, 14.74 gm; m.p. 81-82 °C.

## Procedure for the synthesis *ethyl 6-chloro-4-oxo-1,4-dihydroquinoline-3-carboxylate (2)*

The enamine (1) (~1 gm) was mixed in 5-10 ml of diphenyl ether and irradiated to 250 °C for 1 hour in Anton Paar Monowave 400 microwave reactor. A dark precipitous solution formed within the vial on cooling. 10 ml of diethyl ether or ethyl acetate was added and stirred for an hour. The product was filtered under vacuum, washed with ethyl acetate and dried to obtain the product ethyl 6-chloro-4-oxo-1,4-dihydroquinoline-3-carboxylate (**2**) Yield: 45%.

Conventional thermal cyclization of (**1**) to yield (**2**) was also carried out in an open conical flask. The enamine (**1**) (30 gm, 99.1 mmol) was suspended in 100 ml diphenyl ether and the mixture was stirred and heated to 240-250° C for 2 hour. The reaction was cooled to room temperature, diluted with 200 ml of ethyl acetate and stirred overnight. The residue was filtered, dried and it was re-suspended in boiling ethyl acetate (300 ml) to remove the residual diphenyl ether, cooled to ambient temperature, filtered and dried under vacuum to yield the pure ethyl 6-chloro-4-oxo-1,4-dihydroquinoline-3-carboxylate (**2**). Yield: 88%, 21.87 gm. Creamish white color solid; m.p. >300 °C.

## Procedure for the synthesis of *N-benzyl-6-chloro-4-oxo-1,4-dihydroquinoline-3-carboxamide (3)*

The Ethyl-4-Quinolone-3-Carboxylate (**2**) was suspended in diphenyl ether (4 ml) and Benzylamine (1.2 eq.) was added and the mixture was stirred for an hour at room temperature before being subjected to microwave heating at 210 °C for 1.5 hours, using Anton Paar Monowave 400 microwave reactor. Upon completion the reaction mixture was brought to room temperature and EtOH (~6 ml) was added into the reaction vial and stirred overnight. The mixture was later filtered and the filtrate was dried before being recrystallized using EtOH, to yield N-benzyl-6-chloro-4-oxo-1,4-dihydroquinoline-3-carboxamide (**3**). m.p. >300° C.

## Synthesis of *ethyl 1-allyl-6-chloro-4-oxo-1,4-dihydroquinoline-3-carboxylate (4)*

The Ethyl-4-Quinolone-3-Carboxylate (**2**) (11.9 mmol, 3 gm) was weight and 1.5 times anhydrous K_2_CO_3_ (18 mmol, 2.5 gm) was added. Dry DMF 50 ml was used as solvent. Allyl bromide (14.3 mmol, 1.24 ml) 1.2 ratio, was added dropwise while stirring. Catalytic amount of NaI is added and the reaction was than heated in a reflux assembly at 60-65 °C for 28 hours. The reaction was monitored through TLC and upon completion brought to room temperature and quenched with ice cold water 500ml. The precipitated was filtered and dried to give ethyl 1-allyl-6-chloro-4-oxo-1,4-dihydroquinoline-3-carboxylate (**4**) as a white solid. Yield: 97%, 3.37 gm; m.p. 170-172 °C.

## Synthesis of *1-allyl-6-chloro-4-oxo-1,4-dihydroquinoline-3-carboxylic acid (5)*

Ethyl 1-allyl-6-chloro-4-oxo-1,4-dihydroquinoline-3-carboxylate (**4**) (29.7 mmol, 8.65 gm) was weighed in a flask, THF 100 ml was added and stirred. Later 100 ml of NaOH aqueous solution (NaOH ~4 mole eq, 5.33 gm) was added and the mixture was stirred for 12 hours at room temperature. Monitor the reaction via TLC and upon completion THF was removed under vacuum and the solution was titrated to pH 2-3 using 5 N HCl solution. The precipitate was filtered, washed with water and dried to give 1-allyl-6-chloro-4-oxo-1,4-dihydroquinoline-3-carboxylic acid (**5**) as pinkish white solid. Yield: 96%, 7.49 gm; m.p. 233-235 °C.

## Synthesis of *1-allyl-6-chloro-4-oxo-N-phenyl-1,4-dihydroquinoline-3-carboxamide (6)*

The carboxylic acid **5 (**1.2 mmol, 0.32 gm) was dissolved in 10 ml of anhydrous DMF. Triethylamine 2.5 ratio (3 mmol, 426 µl) was added and the mixture was cooled to 0° C for 20 minutes. Ethyl chloroformate 2 ratio (2.4 mmol, 231 µl) was added dropwise and stirred for 1 hours at 0° C and 1 hour at room temperature. Aniline (2.4 mmol, 222 µl) was added dropwise, the reaction was stirred at 0° C for an hour before being brought to room temperature and stirred for 24 hours. Upon completion the reaction was quenched by pouring into 100 ml ice cold aqueous 0.5 N NaOH solution and stirred vigorously for 6 hours. NaOH solution allowed for the removal of any unreacted starting material or any byproduct carbamate formed thereof. The precipitate formed after addition of NaOH solution was filtered and dried to yield 1-allyl-6-chloro-4-oxo-N-phenyl-1,4-dihydroquinoline-3-carboxamide (**6**). The product was recrystallized with EtOH. White solid, Yield: 91%, 218.0 mg; m.p. 164-165° C.

## Unintended synthesis of *2-hydroxyethyl 1-allyl-6-chloro-4-oxo-1,4-dihydroquinoline-3-carboxylate (7)*

Initially in an attempt to directly couple N-1 substituted 4-Quinolone carboxylic acid (**5**) with the amine we used various solvents and a whole range of reaction condition under microwave to no avail. The reason for this lack of reactivity was the apparent loss of acidity as the N-1 hydrogen has been substituted. However, while all other solvents and reaction conditions did not produce any results, when ethylene glycol was employed as solvent, the small amount of aniline present in the reaction mixture, provided the necessary alkaline conditions for the conversion of carboxylic acid (**5**) to the corresponding ester of **5** and ethylene glycol i.e. 2-hydroxyethyl 1-allyl-6-chloro-4-oxo-1,4-dihydroquinoline-3-carboxylate (**7**). While this is a reported and a very common reaction and is reported to take place in the presence of an alkali or acid. However, we did not use any alkali or acid but supposedly the aniline functioned as the base and facilitated the forward reaction. This may be imply the use of aniline as organo-catalyst for such reactions under similar yet optimized reaction conditions. However, this need further studies to be of any significant consequence.

## Synthesis of 1-allyl-6-chloro-4-oxo-1,4-dihydroquinoline-3-Carboxamides (8-20)

The synthesis of all the remaining 1-allyl-6-chloro-4-oxo-1,4-dihydroquinoline-3-Carboxamides (8-20) was performed as per the above reported procedure for the synthesis of **6** in **Section 2.6**.

# ^1^H-NMR data for Compounds 1-20

| **ID** | **Structure** | **% Yield/ appearance** | **^1^H-NMR** |
| --- | --- | --- | --- |
| **1** |  | 93%  White crystalline | ^1^H-NMR (500 MHz, DMSO-d6): δ 10.70 (1H, d, *J_trans_* = 14.2 Hz, NH), 8.38 (1H, d, *J_trans_* = 14.2 Hz, CH), 7.42-7.49 (4H, m), 4.23 (2H, t, ^3^*J*_HH_ = 7.1 Hz, CH_2_), 4.15 (2H, q, ^3^*J*_HH_ = 7.1 Hz, CH_2_), 1.28 (3H, t, ^3^*J*_HH_ = 7.1 Hz, CH_3_),1.26 (3H, t, ^3^*J_HH_ =*  7.1 Hz, CH_3_).  Mol. Wt. 297.74; m.p. 81-82 ºⅭ. |
| **2** |  | 88%  White crystalline | ^1^H-NMR (300 MHz, DMSO-d6): δ 12.44 (1H, s, NH), 8.58 (1H, s, H-2), 8.08 (1H, d, ^4^*J*_HH_ = 2.5 Hz, H-5), 7.76 (1H, dd, ^4^*J*_HH_ = 2.5 Hz,^3^*J*_HH_ = 8.9 Hz, H-7), 7.67 (1H, d, ^3^*J*_HH_ = 8.9 Hz, H-8), 4.22 (2H, q, ^3^*J*_HH_ = 7.1 Hz, CH_2_), 1.28 (3H, t, ^3^*J*_HH_ = 7.1 Hz, CH_3_).  Mol. Wt. 251.67; m.p. >300 °C. |
| **3** |  | 71%  Shiny white powder | ^1^H-NMR (500 MHz, DMSO- d6): δ 12.92 (1H, *brs,* NH), 10.31 (1H, *brt,* ^3^*J*_HH_ = 5.9 Hz, NH), 8.84 (1H, *s,* H-2), 8.19 (1H, *d,* ^4^*J*_HH_ = 2.5 Hz, H-5), 7.84 (1H, *dd*, ^4^*J*_HH_=2.5 Hz, ^3^*J*_HH_ = 8.8, H-7), 7.78 (1H, *d,* ^3^*J*_HH_ = 8.8 Hz, H-8), 7.36-7.37 (4H, *m,* H-2’, H-3’, H-5’, H-6’), 7.27-7.31 (1H, *m,* H-4’), 4.59 (2H, *d,* ^3^*J*_HH_ = 5.9 Hz, NHCH_2_).  Mol. Wt. 312.75; m.p. 252-253 °C. |
| **4** |  | 97%  White crystalline | ^1^H-NMR (300 MHz, DMSO-d6): δ 8.72 (1H, *s,* H-2), 8.16 (1H, *d,* ^4^*J*_HH_ = 2.4 Hz, H-5), 7.81 (1H, *dd,* ^4^*J*_HH_ = 2.4 Hz,^3^*J*_HH_ = 9.1 Hz, H-7), 7.76 (1H, d, ^3^*J*_HH_ = 9.1 Hz, H-8), 6.05 (1H, *tdd,* ^3^*J*_HH_ = 5.1 Hz, *J*_cis_ = 10.4 Hz, *J*_trans_ = 17.0 Hz, CH’), 5.25 (1H, *d,* *J*_cis_ = 10.4 Hz, CH_2_”), 5.13 (1H, *d,* *J*_trans_ = 17.0 Hz, CH_2_”), 5.06 (2H, *d,* ^3^*J*_HH_ = 5.1 Hz, CH_2_’), 4.24 (2H, *q,* ^3^*J*_HH_ = 7.2 Hz, CH_2_), 1.29 (3H, *t,* ^3^*J*_HH_ = 7.2 Hz, CH_3_),  Mol. Wt. 291.73; m.p. 170-172 °C. |
| **5** |  | 96%  Cream crystalline | ^1^H-NMR (300 MHz, CDCl3): δ 14.58 (1H, s, OH), 8.78 (1H, *s,* H-2), 8.52 (1H, *d,* ^4^*J*_HH_ = 2.5 Hz, H-5), 7.76 (1H, *dd,* ^4^*J*_HH_ = 2.5 Hz, ^3^*J*_HH_ = 9.1 Hz, H-7), 7.55 (1H, *d,* ^3^*J*_HH_ = 9.1 Hz, H-8), 6.03 (1H, *tdd,* ^3^*J*_HH_ = 5.1 Hz, *J*_cis_ = 10.4 Hz, *J*_trans_ = 17.1 Hz, CH’), 5.45 (1H, *d,* *J*_cis_ = 10.4 Hz, CH_2_”), 5.23 (1H, *d,* *J*_trans_ = 17.1 Hz, CH_2_”), 4.94 (2H, *d,* ^3^*J*_HH_ = 5.1 Hz, CH_2_’).  Mol. Wt. 263.68; m.p. 233-235 °C. |
| **6** |  | 91%  White fluffy solid | ^1^H-NMR (300 MHz, CDCl3): δ 12.08 (1H, *s,* NH), 8.86 (1H, s, H-2), 8.53 (1H, d, ^4^*J*_HH_ = 2.5 Hz, H-5), 7.77 (2H, dd, ^4^*J*_HH_ = 1.2 Hz, ^3^*J*_HH_ = 8.7 Hz, H-2’, H-6’), 7.67 (1H, dd, ^4^*J*_HH_ = 2.5 Hz, ^3^*J*_HH_=9.0, H-7), 7.46 (1H, d, ^3^*J*_HH_ = 9.0 Hz, H-8), 7.36 (2 H, dd, ^3^*J*_HH_ = 7.4 Hz, ^3^*J*_HH_ = 8.7 Hz, H-3’, H-5’), 7.12 (1 H, tt, ^4^*J*_HH_ = 1.2 Hz, ^3^*J*_HH_ = 7.4 Hz, H-4’), 6.02 (1H, tdd, ^3^*J*_HH_=5.1 Hz, *J*_cis_=10.5 Hz, *J*_trans_=17.1 Hz, CH’), 5.40 (1H, d, *J*_cis_ = 10.5 Hz, CH_2_”), 5.20 (1H, d, *J*_trans_ = 17.1 Hz, CH_2_”), 4.89 (2H, d, ^3^*J*_HH_ = 5.1 Hz, CH_2_’).  Mol. Wt. 338.79; m.p. 164-165 °C. |
| **7** |  | 20%  Yellowish powder | ^1^H-NMR (500 MHz, DMSO- d6): δ 8.80 (1H, *s,* H-2), 8.19 (1H, *d,* ^4^*J*_HH_ = 2.5 Hz, H-5), 7.85 (1H, *dd*, ^4^*J*_HH_ = 2.5 Hz, ^3^*J*_HH_ = 9.3, H-7), 7.79 (1H, *d,* ^3^*J*_HH_ = 9.3 Hz, H-8), 6.08 (1H, *tdd,* ^3^*J*_HH_ = 5.0 Hz, *J*_cis_= 10.9 Hz, *J*_trans_ = 16.3 Hz, CH’), 5.28 (1H, *d,* *J*_cis_ = 10.9 Hz, CH_2_”), 5.14 (1H, *d,* *J*_trans_ = 16.3 Hz, CH_2_”), 5.08 (2H, *d,* ^3^*J*_HH_ = 5.0 Hz, CH_2_’), 4.89 (1H, *t,* ^3^*J*_HH_ = 5.4 Hz, OH), 4.22 (2H, *t,* ^3^*J*_HH_ = 5.4 Hz, O-CH_2_-), 3.69 (2H, *t,* ^3^*J*_HH_ = 5.4 Hz, -CH_2_-OH).  Mol. Wt. 307.73; m.p. 160-162 °C. |
| **8** |  | 93%  White crystalline | ^1^H-NMR (300 MHz, CDCl3): δ 10.28 (1H, *brt,* ^3^*J*_HH_ = 5.7 Hz, NH), 8.81 (1H, *s,* H-2), 8.48 (1H, *d,* ^4^*J*_HH_ = 2.5 Hz, H-5), 7.64 (1H, *dd*, ^4^*J*_HH_ = 2.5 Hz, ^3^*J*_HH_ = 9.1, H-7), 7.44 (1H, *d,* ^3^*J*_HH_ = 9.1 Hz, H-8), 7.25-7.41 (5H, *m,* H-2”, H-3’, H-4’, H-5’, H-6’), 6.00 (1H, *tdd,* ^3^*J*_HH_ = 5.0 Hz, *J*_cis_ = 10.5 Hz, *J*_trans_ = 17.1 Hz, CH’), 5.38 (1H, *d,* *J*_cis_ = 10.5 Hz, CH_2_”), 5.17 (1H, *d,* *J*_trans_ = 17.1 Hz, CH_2_”), 4.86 (2H, *d,* ^3^*J*_HH_ = 5.0 Hz, CH_2_’), 4.69 (2H, *d,* ^3^*J*_HH_ = 5.7 Hz, NHCH_2_).  Mol. Wt. 352.82; m.p. 160-162 °C. |
| **9** |  | 90%  White crystalline | ^1^H-NMR (300 MHz, CDCl3): δ 12.16 (1H, *s,* NH), 8.85 (1H, *s,* H-2), 8.53 (1H, *d,* ^4^*J*_HH_ = 2.5 Hz, H-5), 7.72 (2H, *d,* ^3^*J*_HH_ = 8.8 Hz, H-2’, H-6’), 7.69 (1H, *dd,* ^4^*J*_HH_ = 2.5 Hz, ^3^*J*_HH_ = 9.3, H-7), 7.48 (1H, *d,* ^3^*J*_HH_ = 9.3 Hz, H-8), 7.32 (2H, *d,* ^3^*J*_HH_ = 8.8 Hz, H-3’, H-5’), 6.03 (1H, *tdd,* ^3^*J*_HH_ = 5.0 Hz, *J*_cis_ = 10.6 Hz, *J*_trans_ = 17.0 Hz, CH’), 5.41 (1H, *d,* *J*_cis_ = 10.6 Hz, CH_2_”), 5.21 (1H, *d,* *J*_trans_ = 17.0 Hz, CH_2_”), 4.90 (2H, *d,* ^3^*J*_HH_ = 5.0 Hz, CH_2_’).  Mol. Wt. 373.23; m.p. 205-206 °C. |
| **10** |  | 90%  White crystalline | ^1^H-NMR (300 MHz, CDCl3): δ 10.31 (1H, *brt,* ^3^*J*_HH_ = 5.9 Hz, NH), 8.80 (1H, *s,* H-2), 8.47 (1H, *d,* ^4^*J*_HH_ = 2.5 Hz, H-5), 7.65 (1H, *dd,* ^4^*J*_HH_ = 2.5 Hz, ^3^*J*_HH_=9.1, H-7), 7.44 (1H, *d,* ^3^*J*_HH_ = 9.1 Hz, H-8), 7.32 (2H, *d,* ^3^*J*_HH_ = 9.0 Hz, H-2’, H-6’), 7.29 (2H, *d,* ^3^*J*_HH_ = 9.0 Hz, H-3’, H-5’), 6.00 (1H, *tdd,* ^3^*J*_HH_ = 5.1 Hz, *J*_cis_=10.4 Hz, *J*_trans_ = 17.0 Hz, CH’), 5.38 (1H, *d,* *J*_cis_ = 10.4 Hz, CH_2_”), 5.18 (1H, *d,* *J*_trans_ = 17.0 Hz, CH_2_”), 4.86 (2H, *d,* ^3^*J*_HH_ = 5.1 Hz, CH_2_’), 4.64 (2H, *d,* ^3^*J*_HH_ = 5.9 Hz, NHCH_2_).  Mol. Wt. 387.26; m.p. 148-149 °C. |
| **11** |  | 89%  White crystalline powder | ^1^H-NMR (300 MHz, CDCl3): δ 12.19 (1H, *brs,* NH), 8.84 (1H, *s,* H-2), 8.53 (1H, *d,* ^4^*J*_HH_ = 2.5 Hz, H-5), 7.94 (1H, *t,* ^4^*J*_HH_ = 2.0 Hz, H-2’), 7.68 (1H, *dd,* ^4^*J*_HH_ = 2.5 Hz, ^3^*J*_HH_ = 9.1, H-7), 7.55 (1H, *dd,* ^4^*J*_HH_ = 2.0 Hz, ^3^*J*_HH_ = 8.0 Hz, H-6’), 7.48 (1H, *d,* ^3^*J*_HH_ = 9.1 Hz, H-8),7.27 (1H, *t,* ^3^*J*_HH_ = 8.1 Hz, H-5’),7.08 (1H, *dd,* ^4^*J*_HH_ = 2.0 Hz, ^3^*J*_HH_ = 8.0 Hz, H-4’), 6.03 (1H, *tdd,* ^3^*J*_HH_ = 5.0 Hz, *J*_cis_=10.5 Hz, *J*_trans_ = 17.1 Hz, CH’), 5.42 (1H, *d,* *J*_cis_ = 10.5 Hz, CH_2_”), 5.21 (1H, *d,* *J*_trans_ = 17.1 Hz, CH_2_”), 4.90 (2H, *d,* ^3^*J*_HH_ = 5.0 Hz, CH_2_’).  Mol. Wt. 373.23; m.p. 205-206 °C. |
| **12** |  | 91%  White fluffy solid | ^1^H-NMR (300 MHz, CDCl3): δ 10.33 (1H, *brt,* ^3^*J*_HH_ = 5.9 Hz, NH), 8.80 (1H, *s,* H-2), 8.48 (1H, *d,* ^4^*J*_HH_ = 2.5 Hz, H-5), 7.66 (1H, *dd,* ^4^*J*_HH_ = 2.5 Hz, ^3^*J*_HH_ = 9.1, H-7), 7.45 (1H, *d,* ^3^*J*_HH_ = 9.1 Hz, H-8), 7.36 (1H, *s,* ^3^*J*_HH_ = 9.0 Hz, H-2’), 7.20-7.27 (3H, *m,* ^3^*J*_HH_ = 9.0 Hz, H-3’, H-6’, H-5’), 6.00 (1H, *tdd,* ^3^*J*_HH_ = 5.1 Hz, *J*_cis_=10.4 Hz, *J*_trans_ = 17.3 Hz, CH’), 5.39 (1H, *d,* *J*_cis_ = 10.4 Hz, CH_2_”), 5.19 (1H, *d,* *J*_trans_ = 17.3 Hz, CH_2_”), 4.87 (2H, *d,* ^3^*J*_HH_ = 5.1 Hz, CH_2_’), 4.66 (2H, *d,* ^3^*J*_HH_ = 5.9 Hz, NHCH_2_).  Mol. Wt. 387.26; m.p. 133-134 °C. |
| **13** |  | 82%  Cream powder | ^1^H-NMR (300 MHz, CDCl3): δ 12.28 (1H, *s,* NH), 8.87 (1H, *s,* H-2), 8.61 (1H, *d,* ^4^*J*_HH_=2.5 Hz, H-5), 8.58 (1H, *dd,* ^4^*J*_HH_ = 1.7 Hz, ^3^*J*_HH_ = 7.7 Hz, H-6’), 7.66 (1H, *dd,* ^4^*J*_HH_=2.5 Hz, ^3^*J*_HH_=9.0, H-7), 7.45 (1H, *d,* ^3^*J*_HH_=9.0 Hz, H-8), 7.08 (1H, *dt,* ^4^*J*_HH_ = 1.7 Hz, ^3^*J*_HH_ = 7.7 Hz, H-5’), 6.99 (1H, *dt,* ^4^*J*_HH_ = 1.5 Hz, ^3^*J*_HH_ = 7.7 Hz, H-4’), 6.95 (1H, *dd,* ^4^*J*_HH_ = 1.5 Hz, ^3^*J*_HH_ = 7.7 Hz, H-3’), 6.02 (1H, *tdd,* ^3^*J*_HH_ = 5.0 Hz, *J*_cis_ = 10.5 Hz, *J*_trans_ = 17.2 Hz, CH’), 5.40 (1H, *d,* *J*_cis_ = 10.5 Hz, CH_2_”), 5.20 (1H, *d,* *J*_trans_ = 17.2 Hz, CH_2_”), 4.89 (2H, *d,* ^3^*J*_HH_ = 5.0 Hz, CH_2_’), 4.03 (3H, *s*, *o*-OCH_3_).  Mol. Wt. 368.82; m.p. 183-185 °C. |
| **14** |  | 90%  Cream crystalline | ^1^H-NMR (300 MHz, CDCl3): δ 10.23 (1H, *brt,* ^3^*J*_HH_ = 5.9 Hz, NH), 8.79 (1H, *s,* H-2), 8.49 (1H, *d,* ^4^*J*_HH_ = 2.5 Hz, H-5), 7.63 (1H, *dd,* ^4^*J*_HH_ = 2.5 Hz, ^3^*J*_HH_ = 9.1, H-7), 7.42 (1H, *d,* ^3^*J*_HH_ = 9.1 Hz, H-8), 7.36 (1H, *dd,* ^4^*J*_HH_ = 1.3 Hz, ^3^*J*_HH_ = 7.4 Hz, H-6’), 7.22 (1H, *dd,* ^4^*J*_HH_ = 1.5 Hz, ^3^*J*_HH_ = 7.9 Hz, H-3’), 6.88-6.94 (2H, *m,* H-5H-4’), 5.98 (1H, *tdd,* ^3^*J*_HH_ = 5.0 Hz, *J*_cis_ = 10.5 Hz, *J*_trans_=17.2 Hz, CH’), 5.36 (1H, *d,* *J*_cis_ = 10.5 Hz, CH_2_”), 5.16 (1H, *d,* *J*_trans_ = 17.2 Hz, CH_2_”), 4.84 (2H, *d,* ^3^*J*_HH_ = 5.0 Hz, CH_2_’), 4.68 (2H, *d,* ^3^*J*_HH_ = 5.9 Hz, NHCH_2_), 3.92 (3H, *s*, *o*-OCH_3_).  Mol. Wt. 368.82; m.p. 183-185 °C. |
| **15** |  | 96%  Dark grey powder | ^1^H-NMR (300 MHz, CDCl_3_): δ 12.11 (1H, *brs,* NH), 8.85 (1H, *s,* H-2), 8.60 (1H, *d,* ^4^*J*_HH_ = 2.5 Hz, H-5), 8.46 (1H, *d,* ^3^*J*_HH_ = 8.8 Hz, H-6’), 7.65 (1H, *dd,* ^4^*J*_HH_ = 2.5 Hz, ^3^*J*_HH_ = 9.1, H-7), 7.45 (1H, *d,* ^3^*J*_HH_ = 9.1 Hz, H-8), 6.55 (1H, *d,* ^4^*J*_HH_ = 2.6 Hz, H-3’), 6.51 (1H, *dd,* ^4^*J*_HH_ = 2.6 Hz, ^3^*J*_HH_=8.8 Hz, H-5’), 6.02 (1H, *tdd,* ^3^*J*_HH_ = 5.1 Hz, *J*_cis_ = 10.3 Hz, *J*_trans_ = 17.1 Hz, CH’), 5.39 (1H, *d,* *J*_cis_ = 10.3 Hz, CH_2_”), 5.20 (1H, *d,* *J*_trans_ = 17.1 Hz, CH_2_”), 4.88 (2H, *d,* ^3^*J*_HH_ = 5.1 Hz, CH_2_’), 4.00 (3H, *s*, OCH_3_), 3.82 (3H, *s*, OCH_3_).  Mol. Wt. 398.84; m.p. b/w 180-210 °C turns too dark to be observable |
| **16** |  | 89%  Fluffy white solid | ^1^H-NMR (300 MHz, CDCl_3_): δ 10.16 (1H, *brt,* ^3^*J*_HH_ = 5.8 Hz, NH), 8.78 (1H, *s,* H-2), 8.48 (1H, *d,* ^4^*J*_HH_ = 2.5 Hz, H-5), 7.62 (1H, *dd,* ^4^*J*_HH_ = 2.5 Hz, ^3^*J*_HH_ = 9.1, H-7), 7.41 (1H, *d,* ^3^*J*_HH_ = 9.1 Hz, H-8), 7.26-7.29 (1H, *d,* H-6’ and CHCl_3_), 6.47 (1H, *d,* ^4^*J*_HH_ = 2.4 Hz, H-3’), 6.43 (1H, *dd,* ^4^*J*_HH_ = 2.4 Hz, ^3^*J*_HH_=8.2 Hz, H-5’), 5.98 (1H, *tdd,* ^3^*J*_HH_ = 5.1 Hz, *J*_cis_ = 10.7 Hz, *J*_trans_ = 17.1 Hz, CH’), 5.35 (1H, *d,* *J*_cis_ = 10.7 Hz, CH_2_”), 5.14 (1H, *d,* *J*_trans_ = 17.1 Hz, CH_2_”), 4.83 (2H, *d,* ^3^*J*_HH_ = 5.1 Hz, CH_2_’), 4.60 (2H, *d,* ^3^*J*_HH_ = 5.8 Hz, NHCH_2_), 3.89 (3H, *s*, OCH_3_), 3.79 (3H, *s*, OCH_3_).  Mol. Wt. 412.87; m.p. 268-270 °C. |
| **17** |  | 94%  Beige powder | ^1^H-NMR (300 MHz, DMSO- d6): δ 11.95 (1H, *s,* OH), 9.28 (1H, s, NH), 9.00 (1H, *s,* H-2), 8.32 (1H, *m,* H-5), 7.90-7.91 (2H, *m,* H-7, H-8), 7.52 (2H, *d,* ^3^*J*_HH_ = 8.8 Hz, H-2’, H-6’), 6.76 (2H, *d,* ^3^*J*_HH_ = 8.8 Hz, H-3’, H-5’), 6.08 (1H, *tdd,* ^3^*J*_HH_ = 5.0 Hz, *J*_cis_ = 10.6 Hz, *J*_trans_ = 16.9 Hz, CH’), 5.27 (1H, *d,* ^1^*J*_HH_ = 0.9 Hz, *J*_cis_ = 10.6 Hz, CH_2_’), 5.21 (2H, *d,* ^3^*J*_HH_ = 5.0 Hz, CH_2_’), 5.15 (1H, *d*, ^1^*J*_HH_ = 0.9 Hz, *J*_trans_ = 16.9 Hz, CH_2_’).  Mol. Wt. 354.79; m.p. 257-259 °C. |
| **18** |  | 96%  Fluffy white | ^1^H-NMR (300 MHz, DMSO- d6): δ 10.09 (1H, *brt,* ^3^*J*_HH_ = 5.8 Hz, NH), 9.33 (1H, *s,* OH), 8.24 (1H, *s,* H-2), 7.86-7.87 (2H, *m,* H-7, H-8), 7.16 (2H, *d,* ^3^*J*_HH_ = 8.6 Hz, H-3’, H-5’), 6.73 (2H, *d,* ^3^*J*_HH_ = 8.6 Hz, H-2’, H-6’), 6.06 (1H, *tdd,* ^3^*J*_HH_ = 5.0 Hz, *J*_cis_ = 10.4 Hz, *J*_trans_ = 17.1 Hz, CH’), 5.26 (1H, *d,* ^1^*J*_HH_ = 1.1 Hz, *J*_cis_ = 10.4 Hz, CH_2_’), 5.17 (2H, *d,* ^3^*J*_HH_ = 5.0 Hz, CH_2_’), 5.12 (1H, *d*, ^1^*J*_HH_ = 1.1 Hz, *J*_trans_ = 17.1 Hz, CH_2_’), 4.44 (2H, *d,* ^3^*J*_HH_ = 5.8 Hz, NHCH_2_).  Mol. Wt. 354.79; m.p. 257-259 °C. |
| **19** |  | 88%  Fluffy white | ^1^H-NMR (300 MHz, CDCl_3_): δ 12.36 (1H, s, NH), 8.85 (1H, *s*, H-2), 8.59 (1H, *d*, ^4^*J*_HH_ = 2.5 Hz, H-5), 8.51 (1H, *dt*, ^4^*J*_HH_ = 1.6 Hz,^3^*J*_HH_ = 8 Hz, H-4’), 7.67 (1H, *dd*, ^4^*J*_HH_ = 2.5 Hz,^3^*J*_HH_ = 9.1 Hz, H-7), 7.47 (1H, *d*, ^3^*J*_HH_ = 9.1 Hz, H-8), 7.13-7.18 (2H, *m*, H-5’, H-6’), 7.02-7.10 (1H, *m*, H-3’), 6.03 (1H, *tdd,* ^3^*J*_HH_ = 5.0 Hz, *J*_cis_ = 10.4 Hz, *J*_trans_ = 17.2 Hz, CH’), 5.41 (1H, *d,* *J*_cis_ = 10.4 Hz, CH_2_”), 5.21 (1H, *d,* *J*_trans_ = 17.2 Hz, CH_2_”), 4.90 (2H, *d,* ^3^*J*_HH_ = 5.0 Hz, CH_2_’).  Mol. Wt. 356.78; m.p. 186-187 °C. |
| **20** |  | 92%  Cream crystalline | ^1^H-NMR (300 MHz, CDCl_3_): δ 10.28 (1H, *brt,* ^3^*J*_HH_ = 5.7 Hz, NH), 8.80 (1H, *s*, H-2), 8.49 (1H, *d*, ^4^*J*_HH_ = 2.5 Hz, H-5), 7.64 (1H, *dd*, ^4^*J*_HH_ = 2.5 Hz,^3^*J*_HH_ = 9.1 Hz, H-7), 7.40-7.45 (2H, *m*, H-8, H-6’), 7.20-7.26 (1H, *m*, H-5’), 7.02-7.12 (2H, *m*, H-4’, H-3’), 5.99 (1H, *tdd,* ^3^*J*_HH_ = 5.0 Hz, *J*_cis_ = 10.5 Hz, *J*_trans_=17.2 Hz, CH’), 5.37 (1H, *d,* *J*_cis_ = 10.5 Hz, CH_2_”), 5.17 (1H, *d,* *J*_trans_ = 17.2 Hz, CH_2_”), 4.85 (2H, *d,* ^3^*J*_HH_ = 5.0 Hz, CH_2_’), 4.73 (2H, *d,* ^3^*J*_HH_ = 5.7 Hz, NHCH_2_).  Mol. Wt. 370.81; m.p. 193-194 °C. |


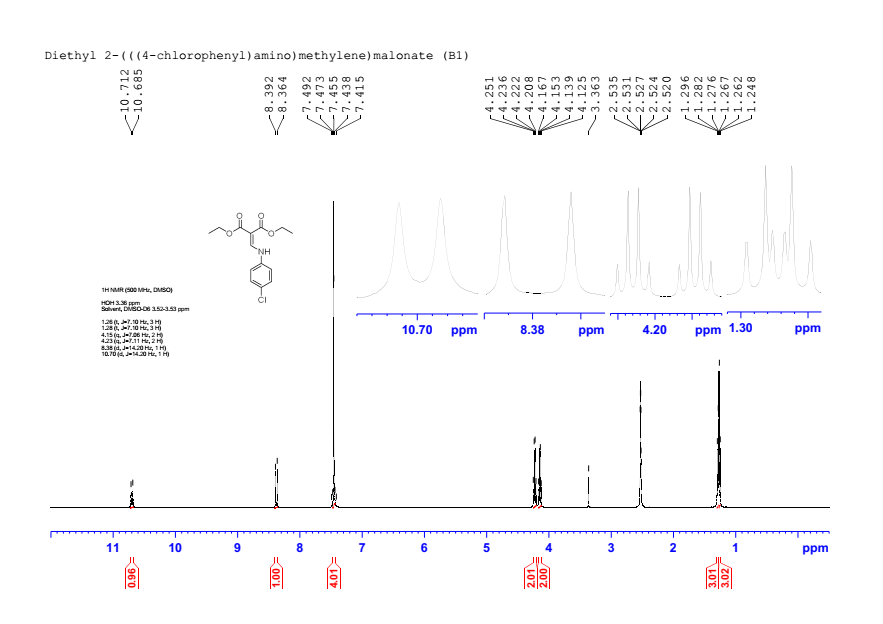


### Figure S1: ^1^H-NMR Spectra of 1


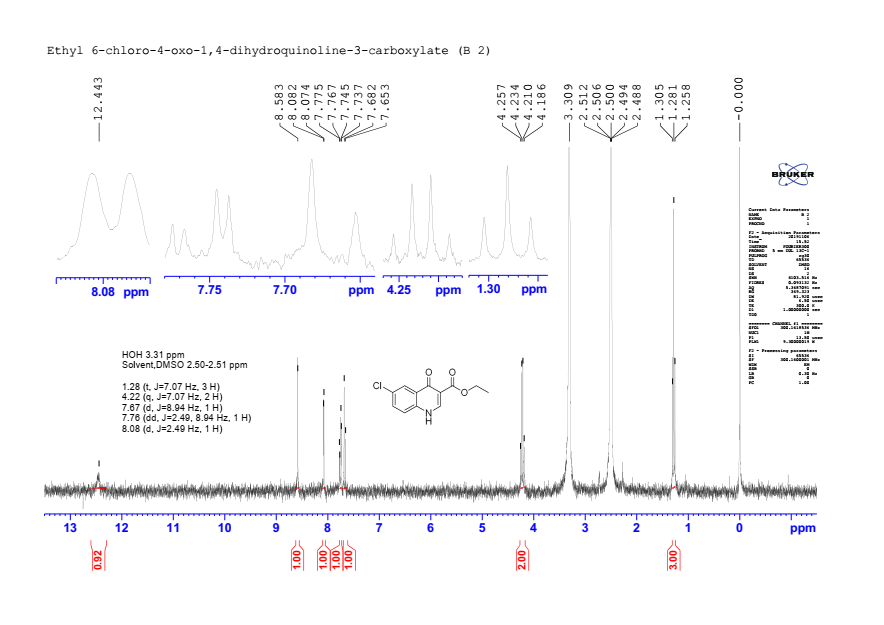


### Figure S2: ^1^H-NMR Spectra of 2

###
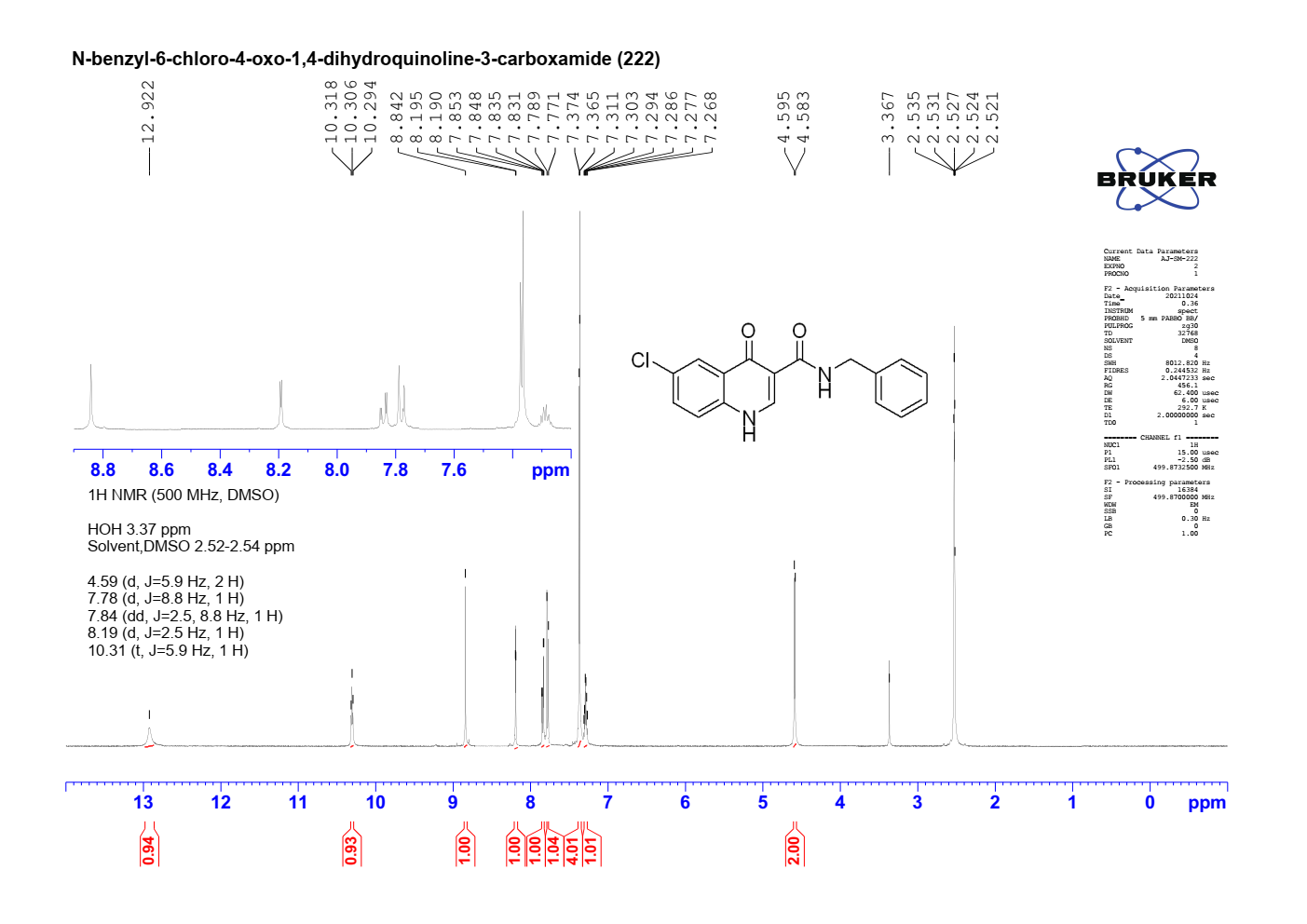


### Figure S3: ^1^H-NMR Spectra of 3


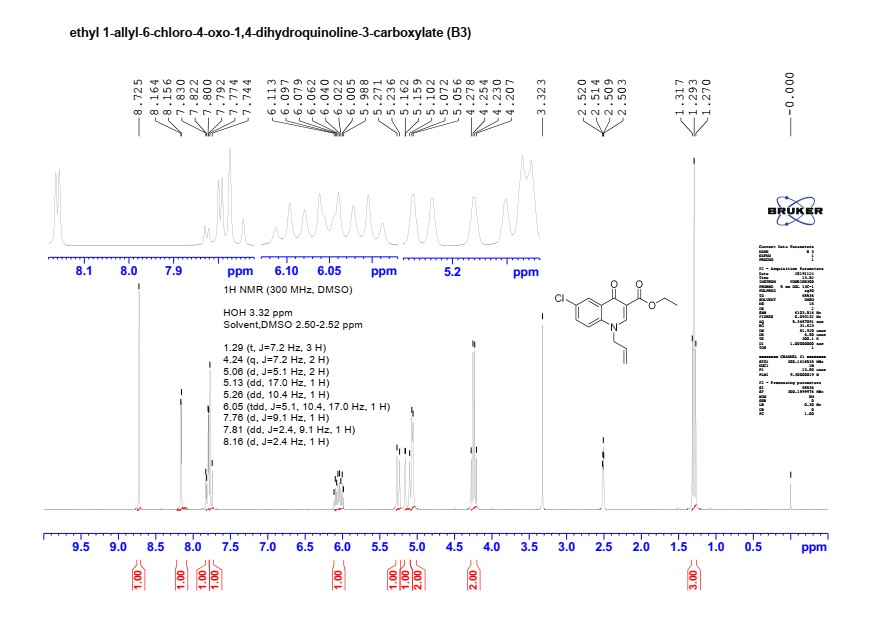


### Figure S4: ^1^H-NMR Spectra of 4


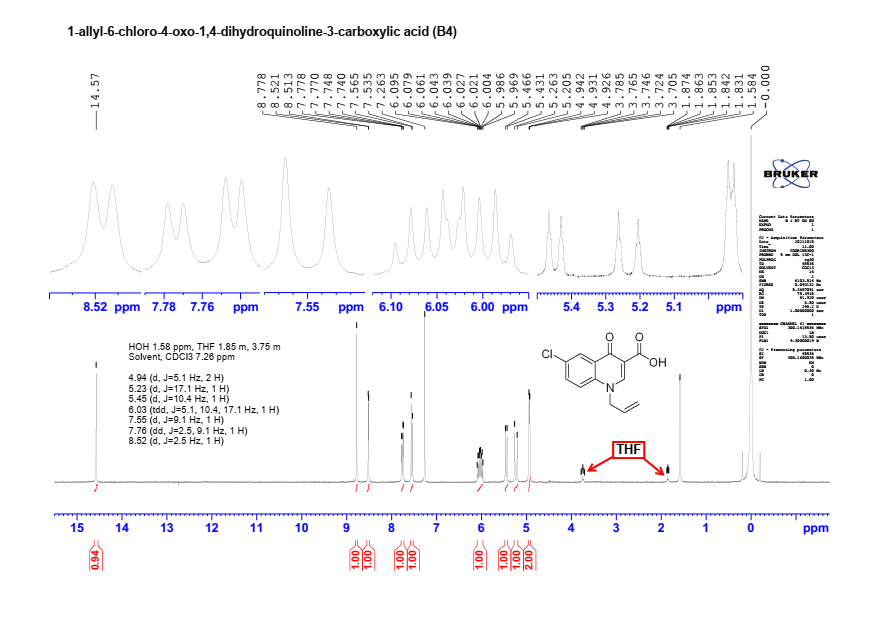


### Figure S5: ^1^H-NMR Spectra of 5


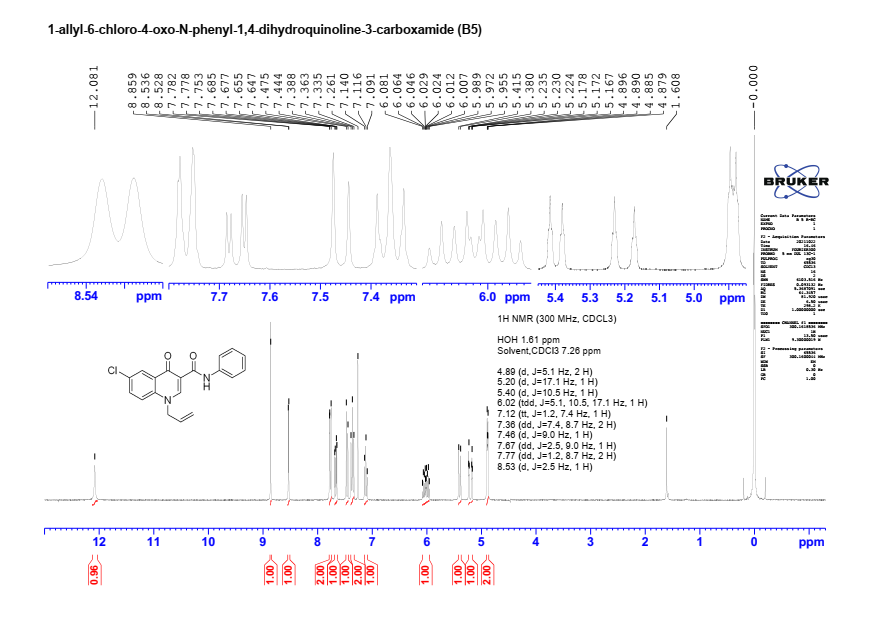


### Figure S6: ^1^H-NMR Spectra of 6


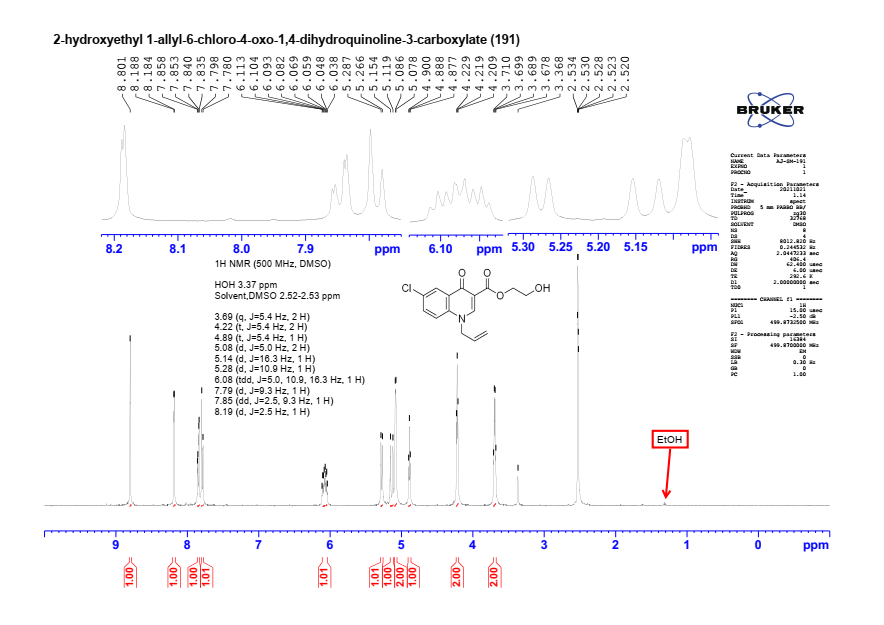


### Figure S7: ^1^H-NMR Spectra of 7


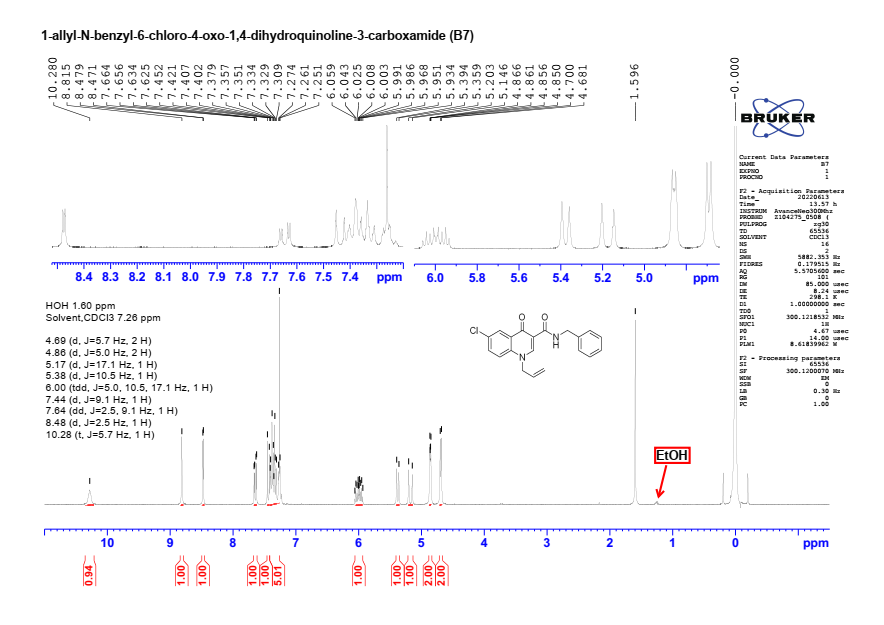


### Figure S8: ^1^H-NMR Spectra of 8


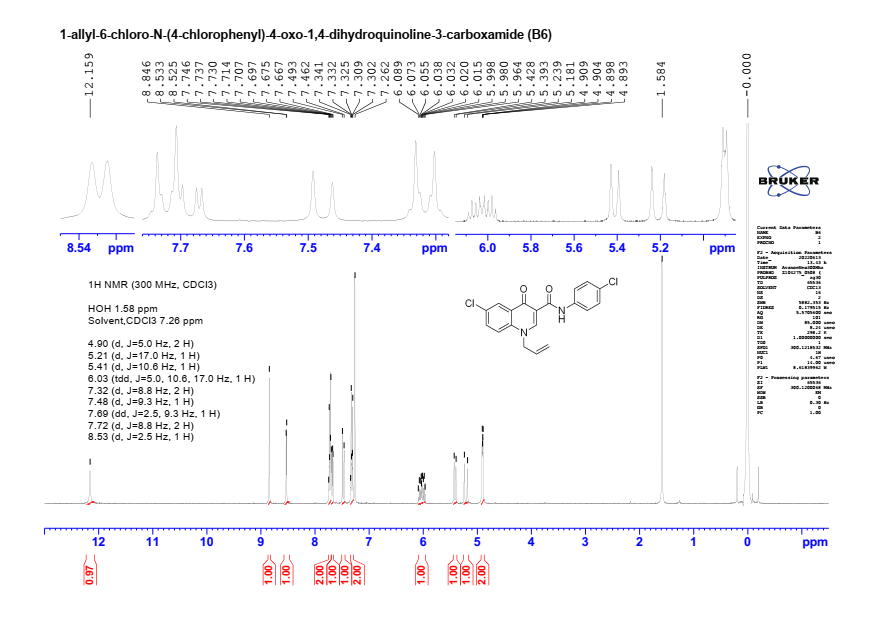


### Figure S9: ^1^H-NMR Spectra of 9


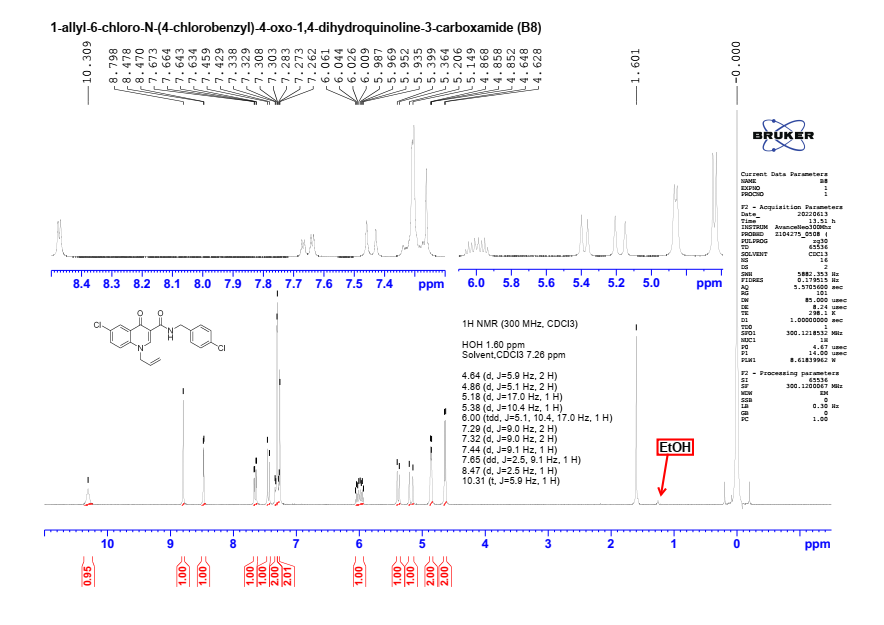


### Figure S10: ^1^H-NMR Spectra of 10


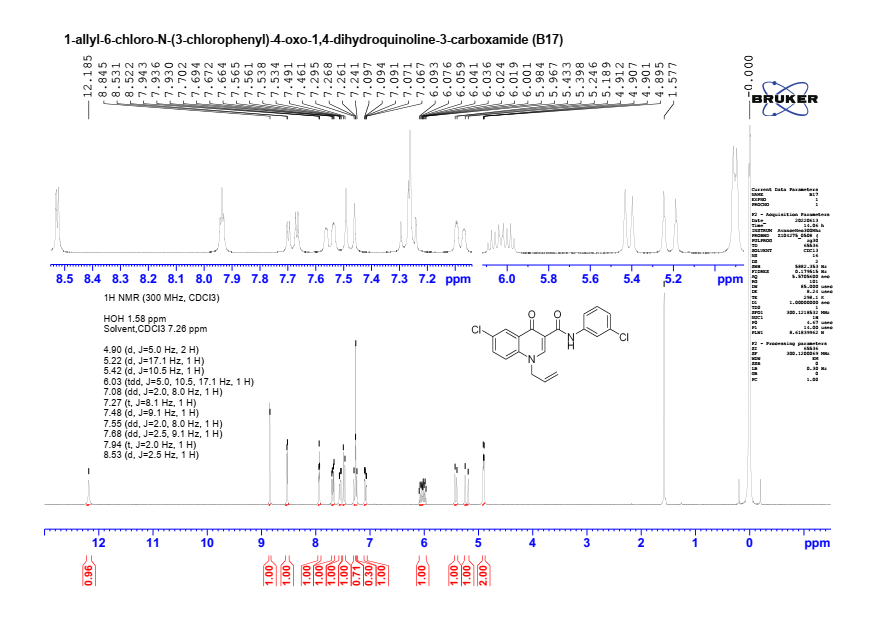


### Figure S11: ^1^H-NMR Spectra of 11


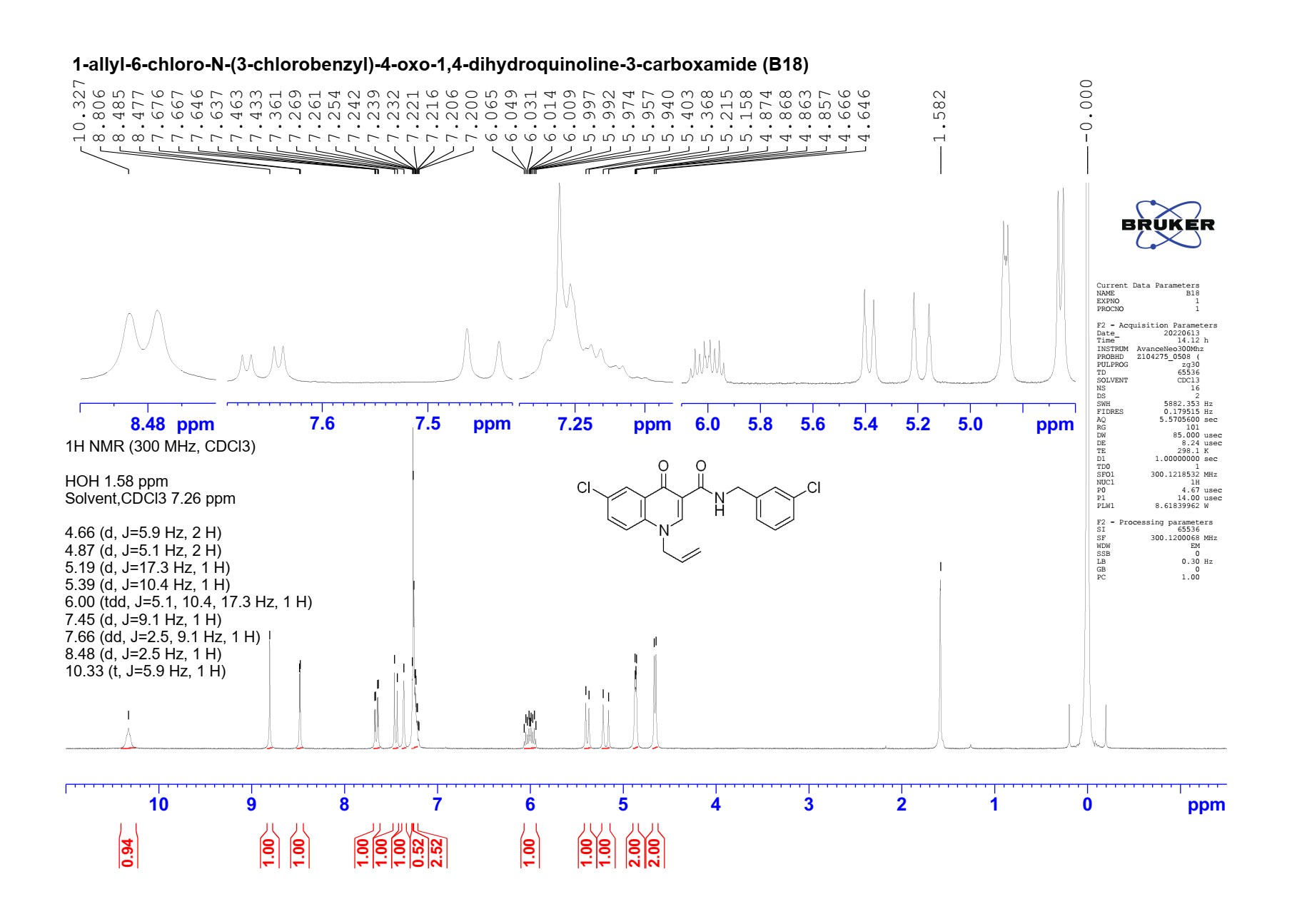


### Figure S12: ^1^H-NMR Spectra of 12


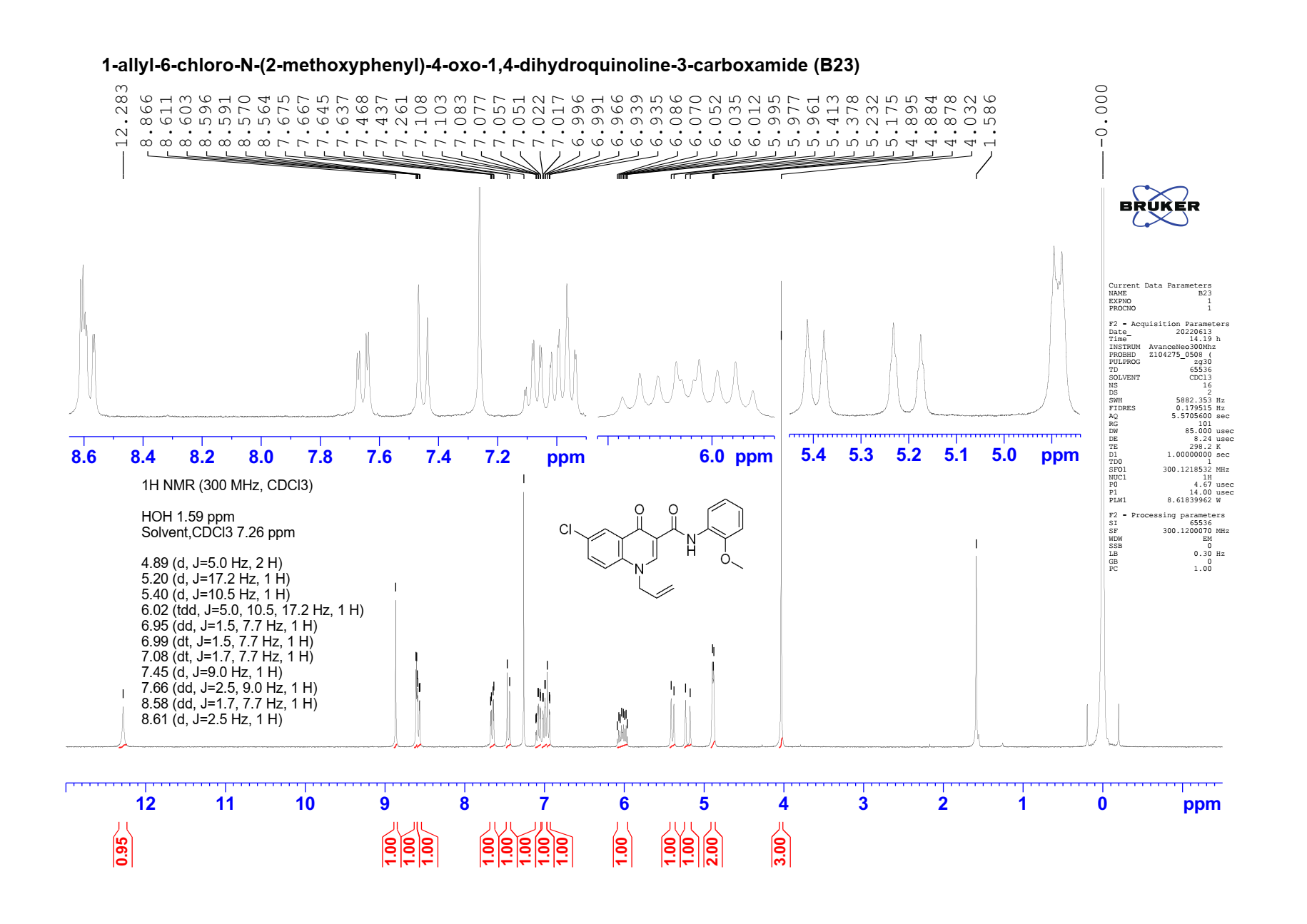


### Figure S13: ^1^H-NMR Spectra of 13


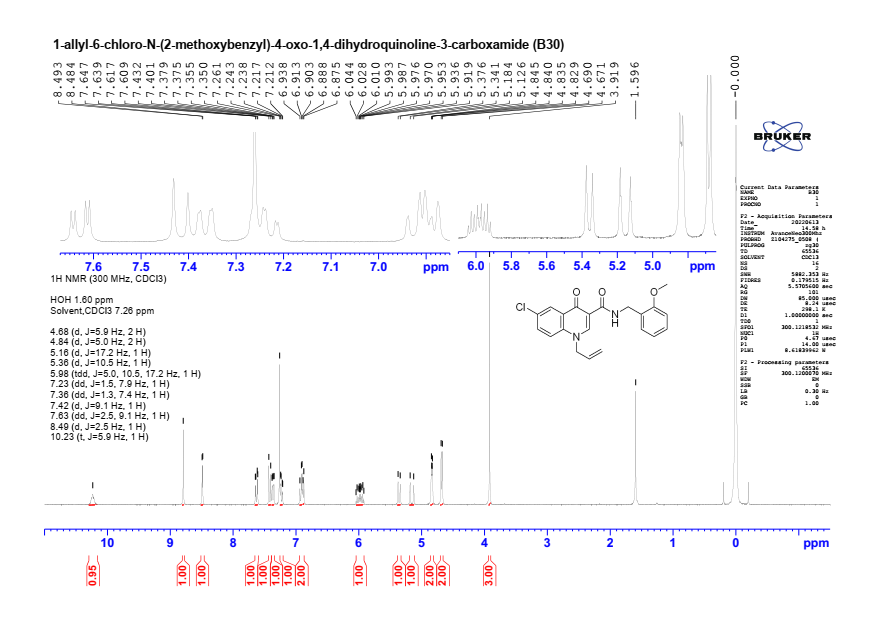


### Figure S14: ^1^H-NMR Spectra of 14


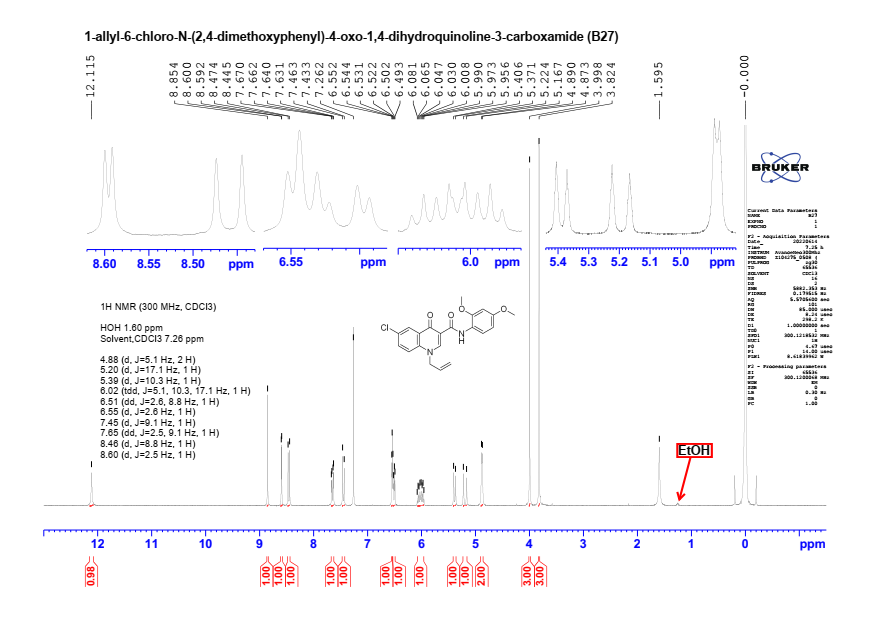


### Figure S15: ^1^H-NMR Spectra of 15


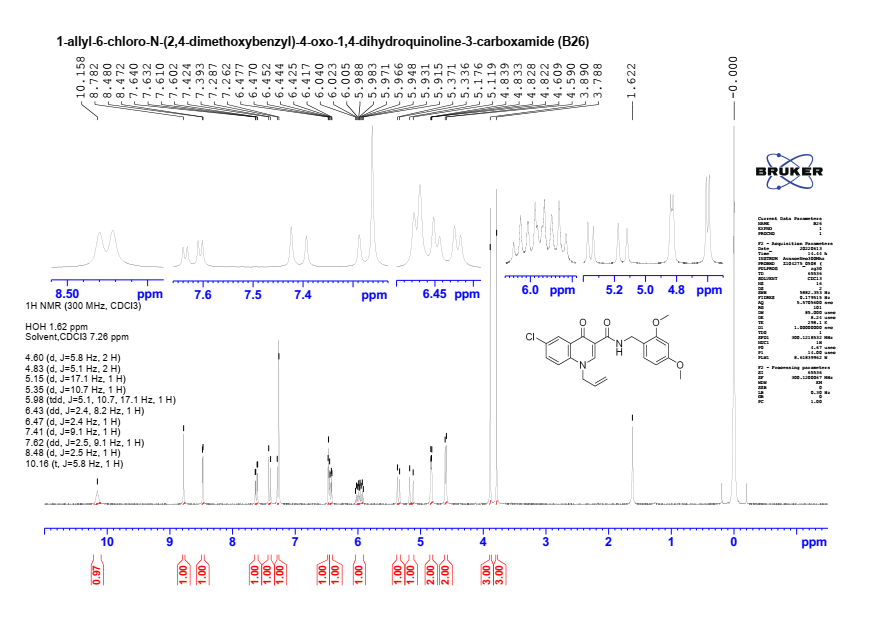


### Figure S16: ^1^H-NMR Spectra of 16


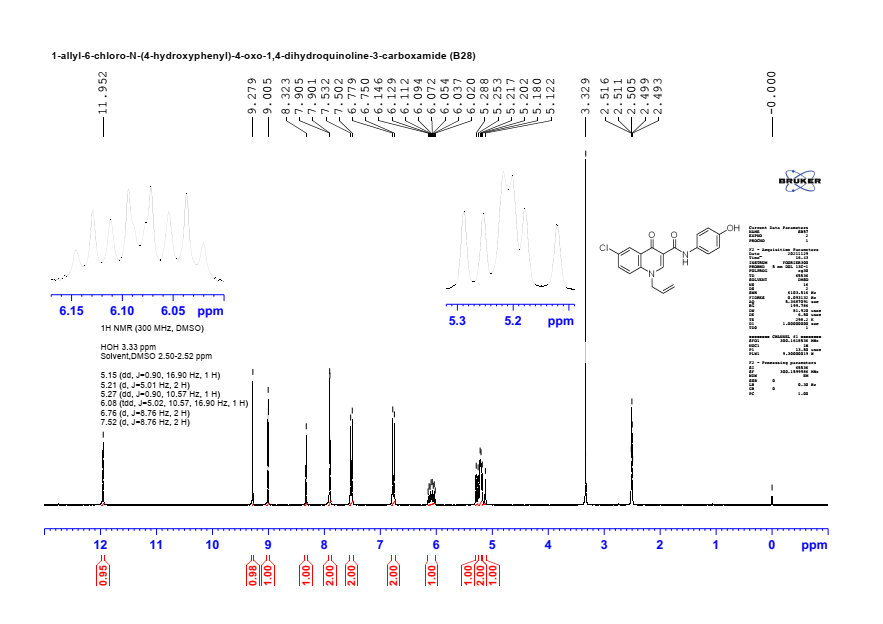


### Figure S17: ^1^H-NMR Spectra of 17


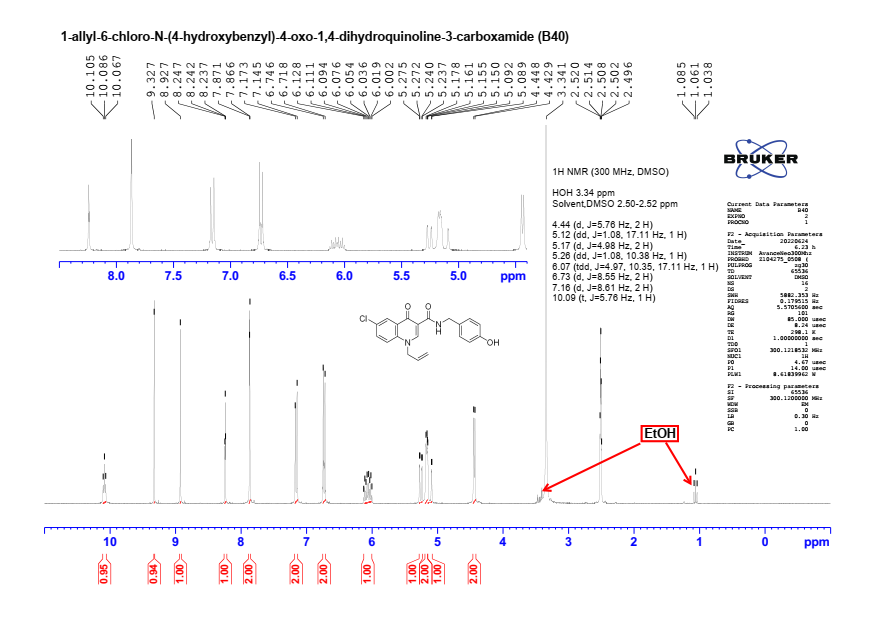


### Figure S18: ^1^H-NMR Spectra of 18

###
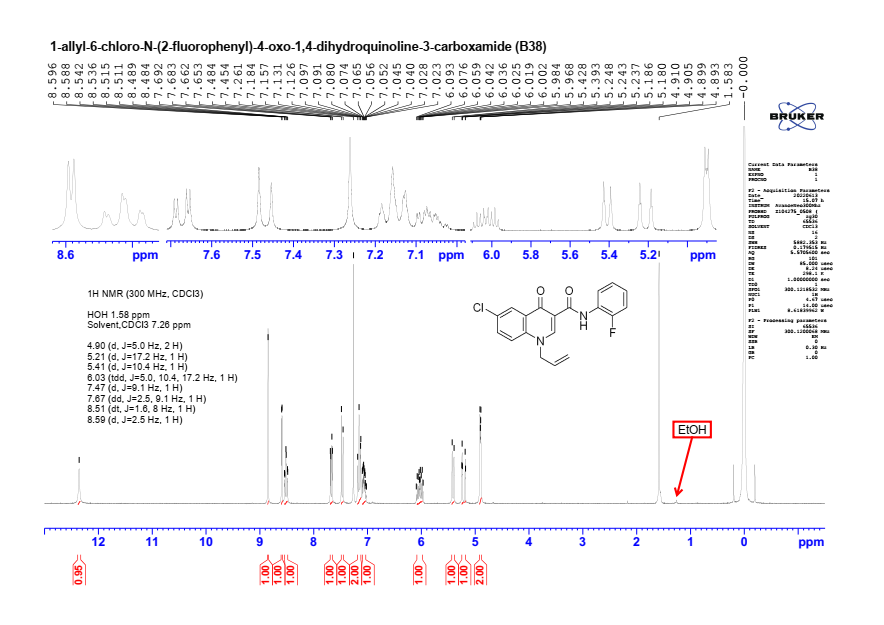
Figure S19: ^1^H-NMR Spectra of 19


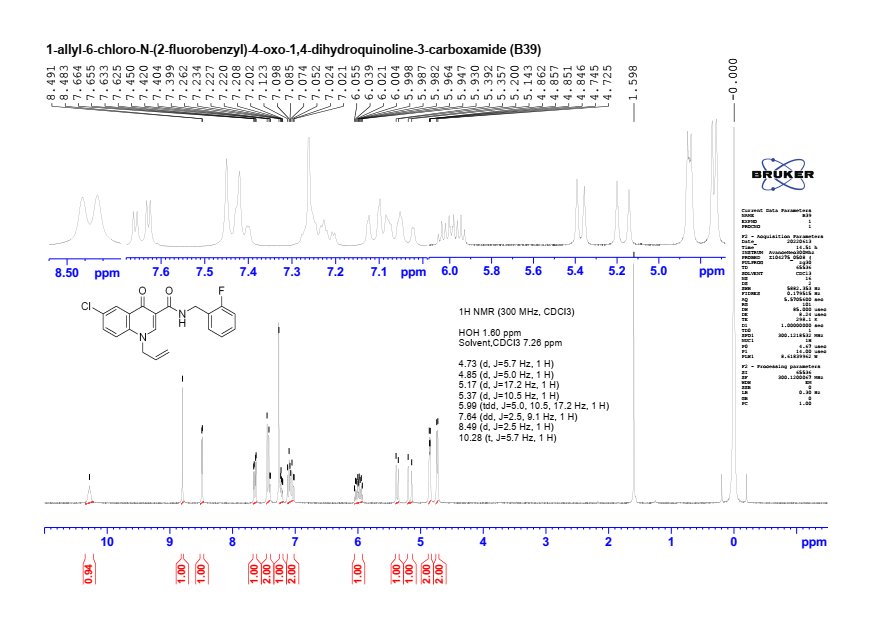


### Figure S20: ^1^H-NMR Spectra of 20

## LCMS analysis methodology

Retention times for liquid chromatography were recorded with an Agilent Infinity Lab LC/MSD equipped with Agilent 1260 Infinity II equipped with DAD WR detector. Chromatography was performed using a 0.65 mL/min flow rate at 40 °C and Acquity UPLC ethylene-bridged hybrid (BEH) C18 (2.1 × 50 mm2, 1.7 μm) column fitted with a C18 guard cartridge. Two mobile phase solutions were used: solution A was 0.1% formic acid in water and solution B was acetonitrile. The method was run with the elution program consisting of a linear gradient starting at 98% A for 0.5 min following injection to 2% A for over 6.5 minutes and holding for 2 additional minutes at 2% A with a total run time of 10 minutes.

## HPLC analysis methodology

Samples were dissolved in Acetonitrile and passed through nylon filter to remove any residue. The samples were injected (5-μL) into Agilent HPLC 1200 operating at a pressure up to 400 bar. The samples were eluted through Zorbax Eclips XDB-C18 Column (narrow bore 3.5-μm. 2.1 × 150 mm) maintained at 40 °C. The samples were eluted using Acetonitrile: Water (60:40) mobile phase at a flow rate of 0.2 ml/min, for a period of 25 minutes per sample. Finally the detection was done using diode array detector (DAD) at 254, 4 nm.

### HPLC/LCMS Data Table

| **ID** | **Retention Time (mins)** | **%age Purity HPLC** | **%age Purity LCMS** |
| --- | --- | --- | --- |
| 3 | 5.174 | - | 89.27 |
| 4 | 3.038 | 99.3882 | - |
| 5 | 4.723 | 99.9553 | - |
| 6 | 12.373 | 99.9655 | - |
| 7 | 4.091 | - | 98.67 |
| 8 | 8.543 | 99.9410 | - |
| 9 | 3.709 | 96.4783 | - |
| 10 | 7.372 | 99.9526 | - |
| 11 | 16.724 | 99.6724 | - |
| 12 | 14.209 | 99.9429 | - |
| 13 | 15.439 | 99.9202 | - |
| 14 | 5.228 | 99.5384 | - |
| 15 | 5.422 | 99.3382 | - |
| 16 | 4.941 | 99.6874 | - |
| 17 | 3.255 | 100.0000 | - |
| 18 | 2.898 | 100.0000 | - |
| 19 | 7.931 | 100.0000 | - |
| 20 | 5.229 | 100.0000 | - |


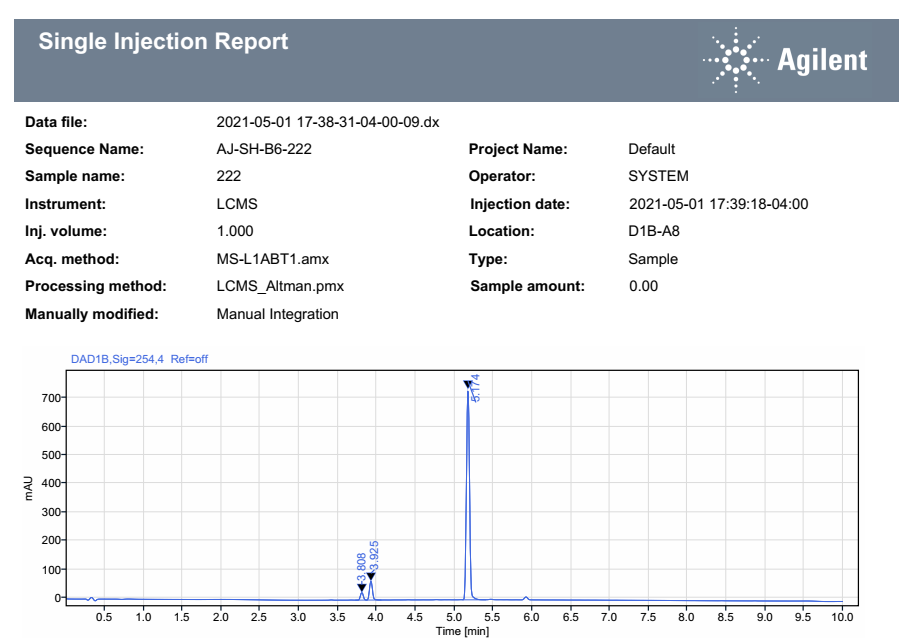

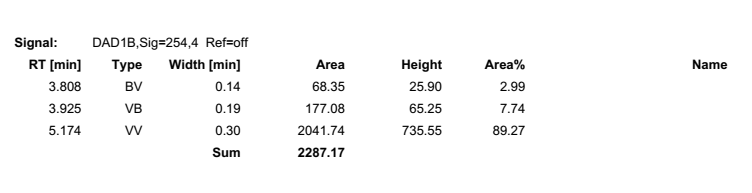


### Figure S21a: LCMS Data of 3


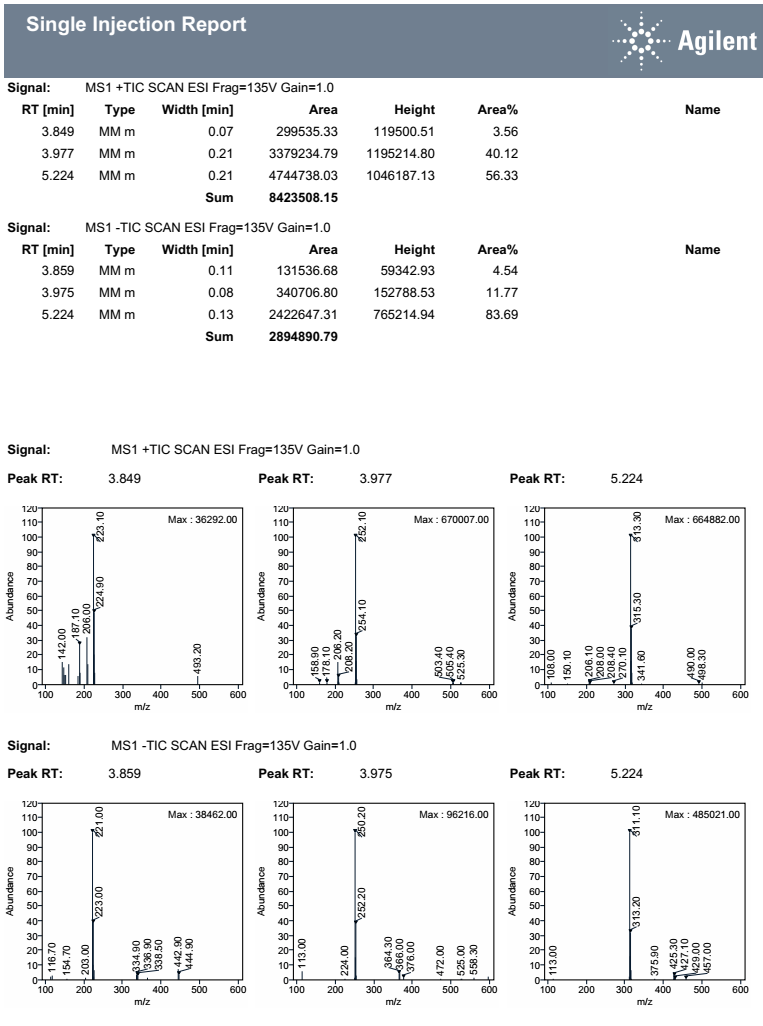


### Figure S21b: MS Data plot of 3


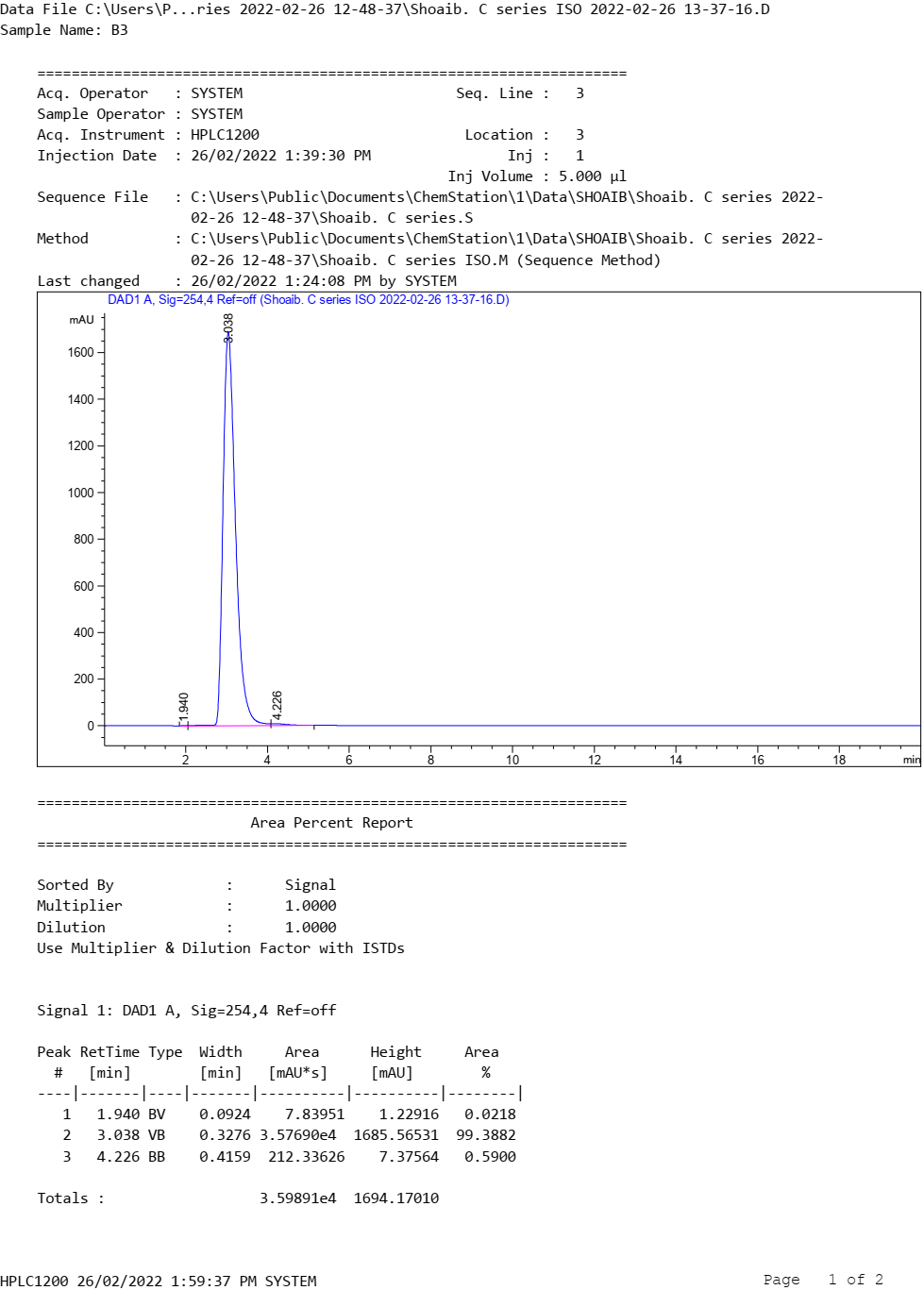


### Figure S22: HPLC Data of 4


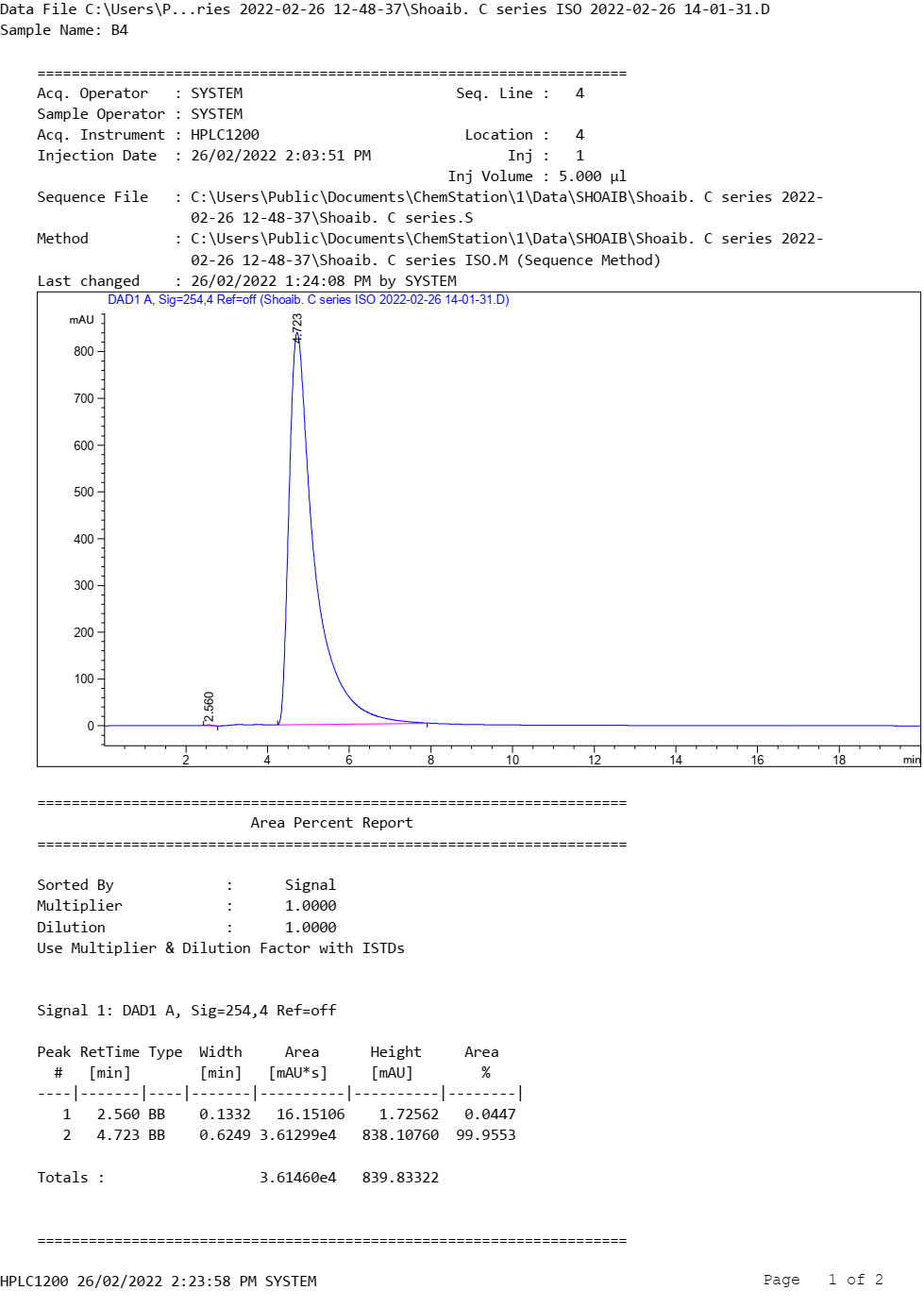


### Figure S23: HPLC Data of 5


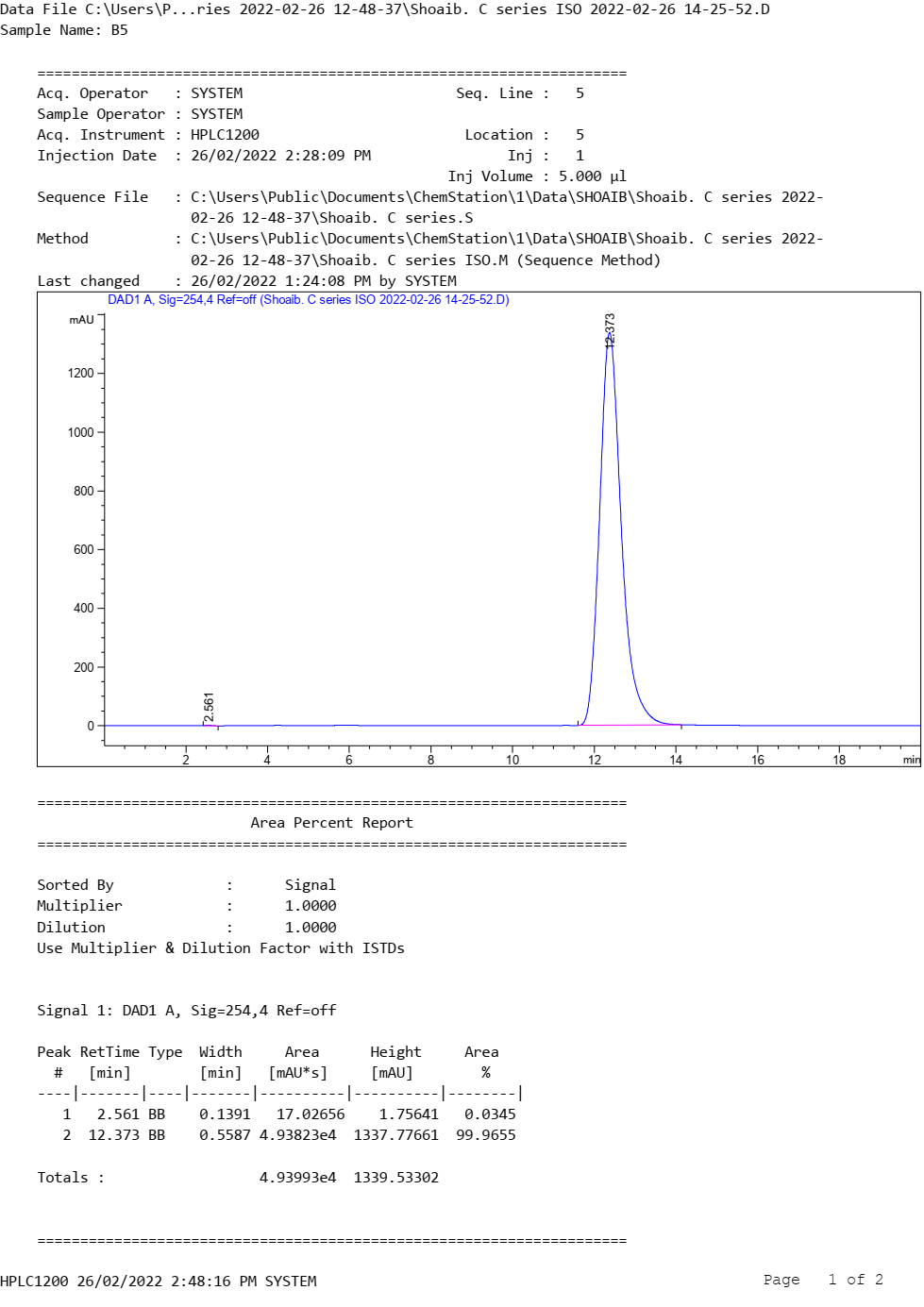


### Figure S24: HPLC Data of 6

###
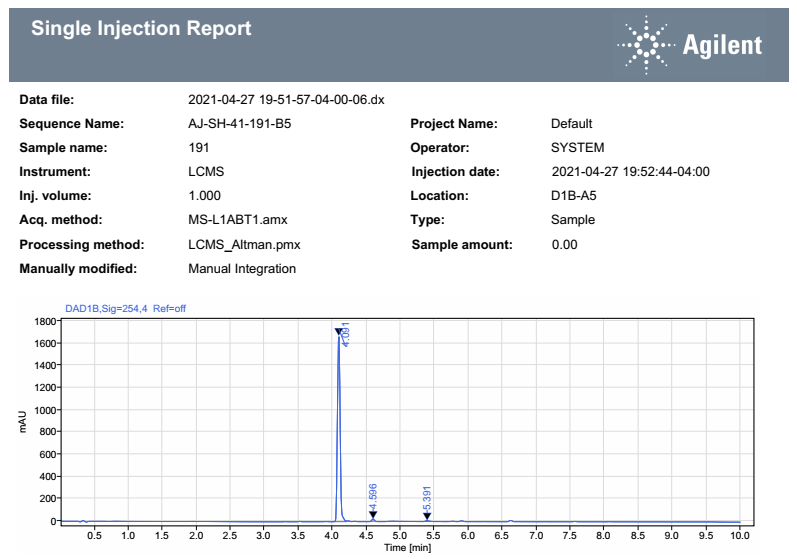

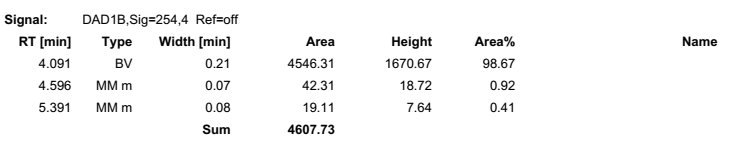


### Figure S25a: LCMS Data of 7


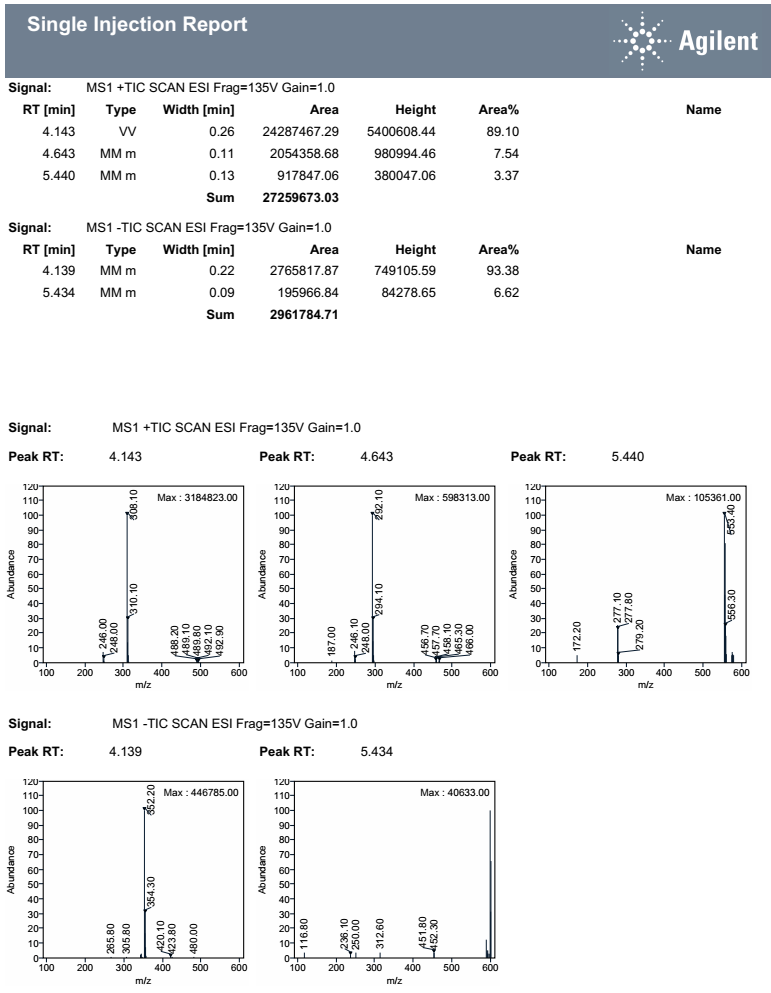


### Figure S25b: MS Data plot of 7


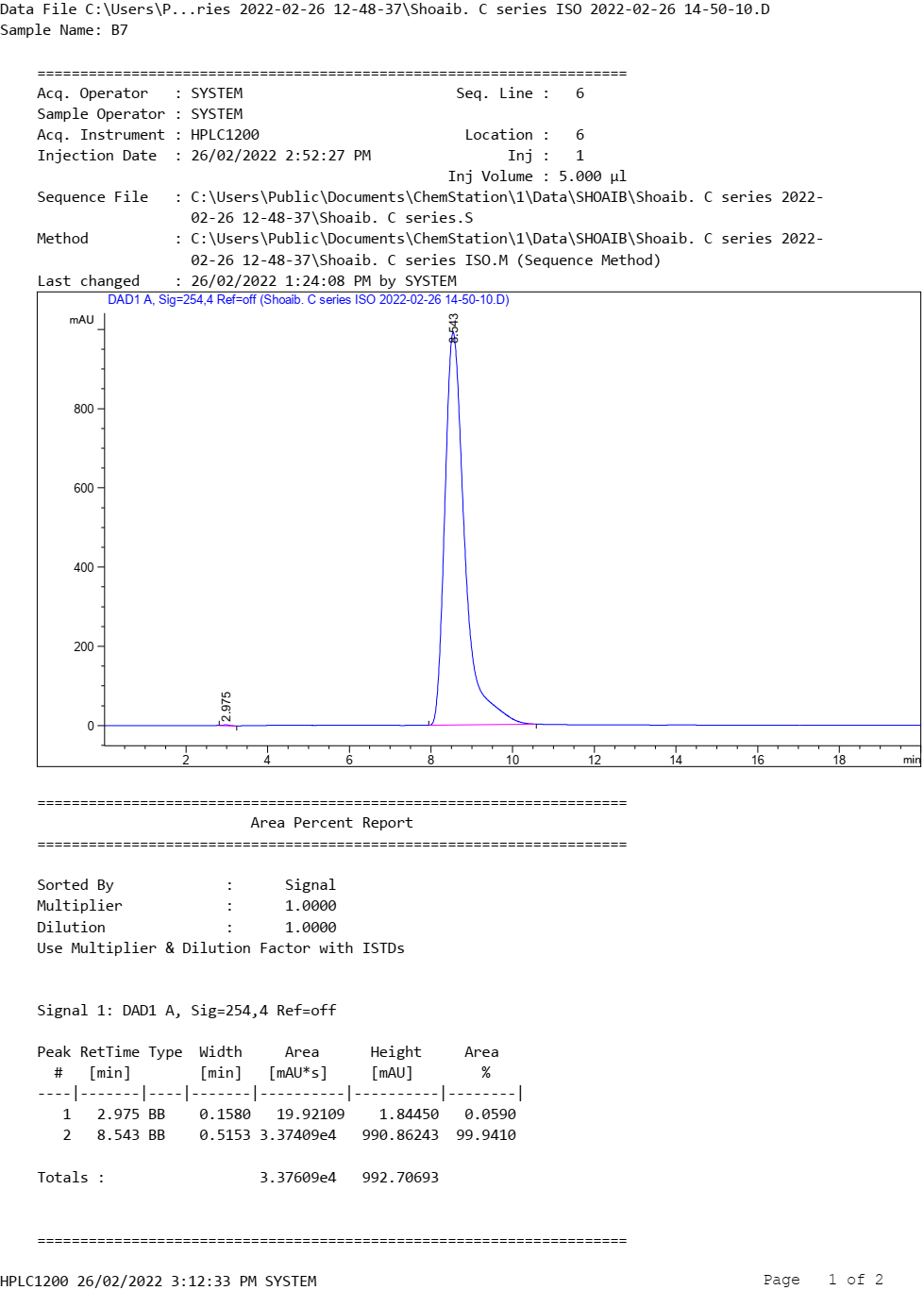


### Figure S26: HPLC Data of 8


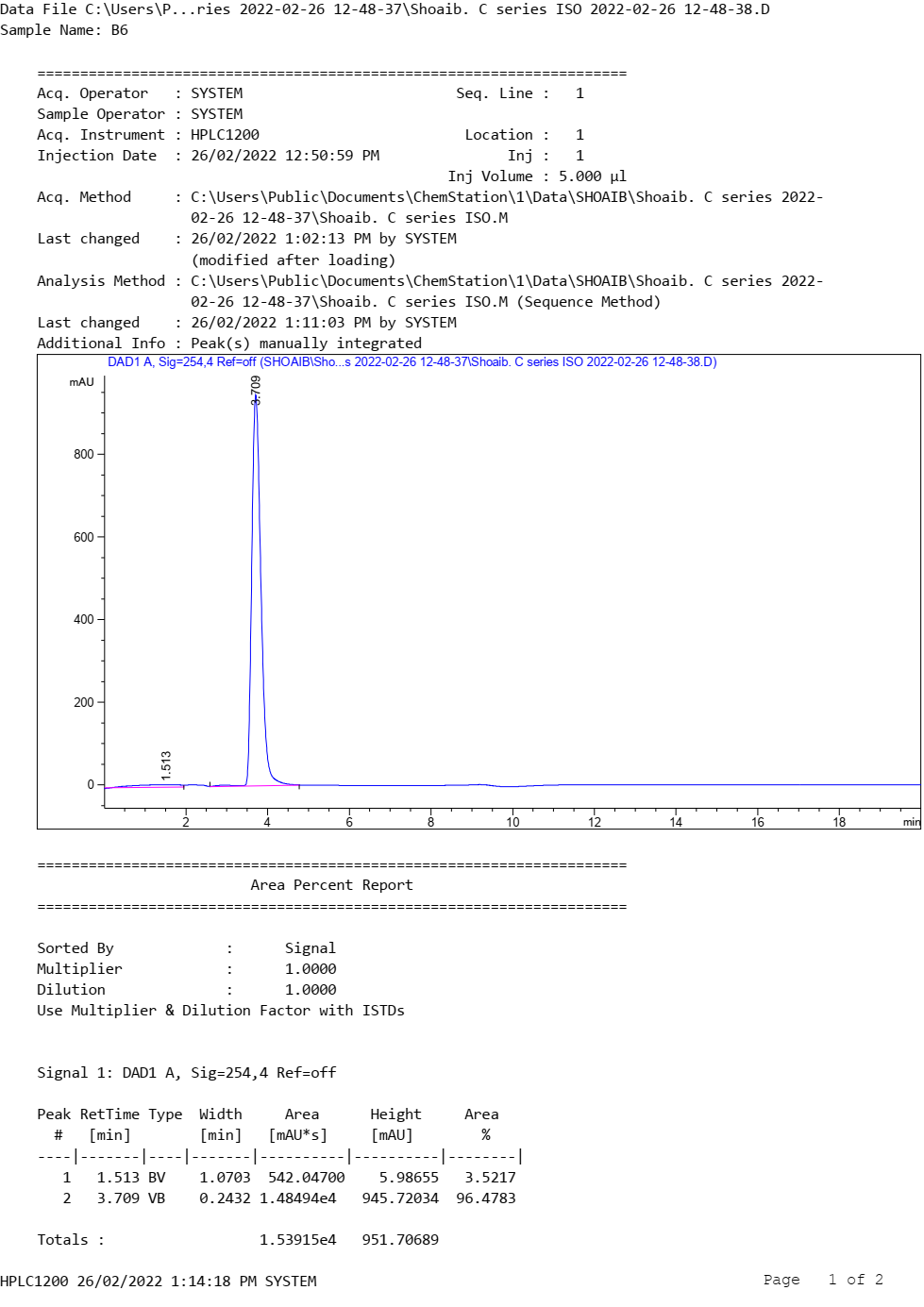


### Figure S27: HPLC Data of 9


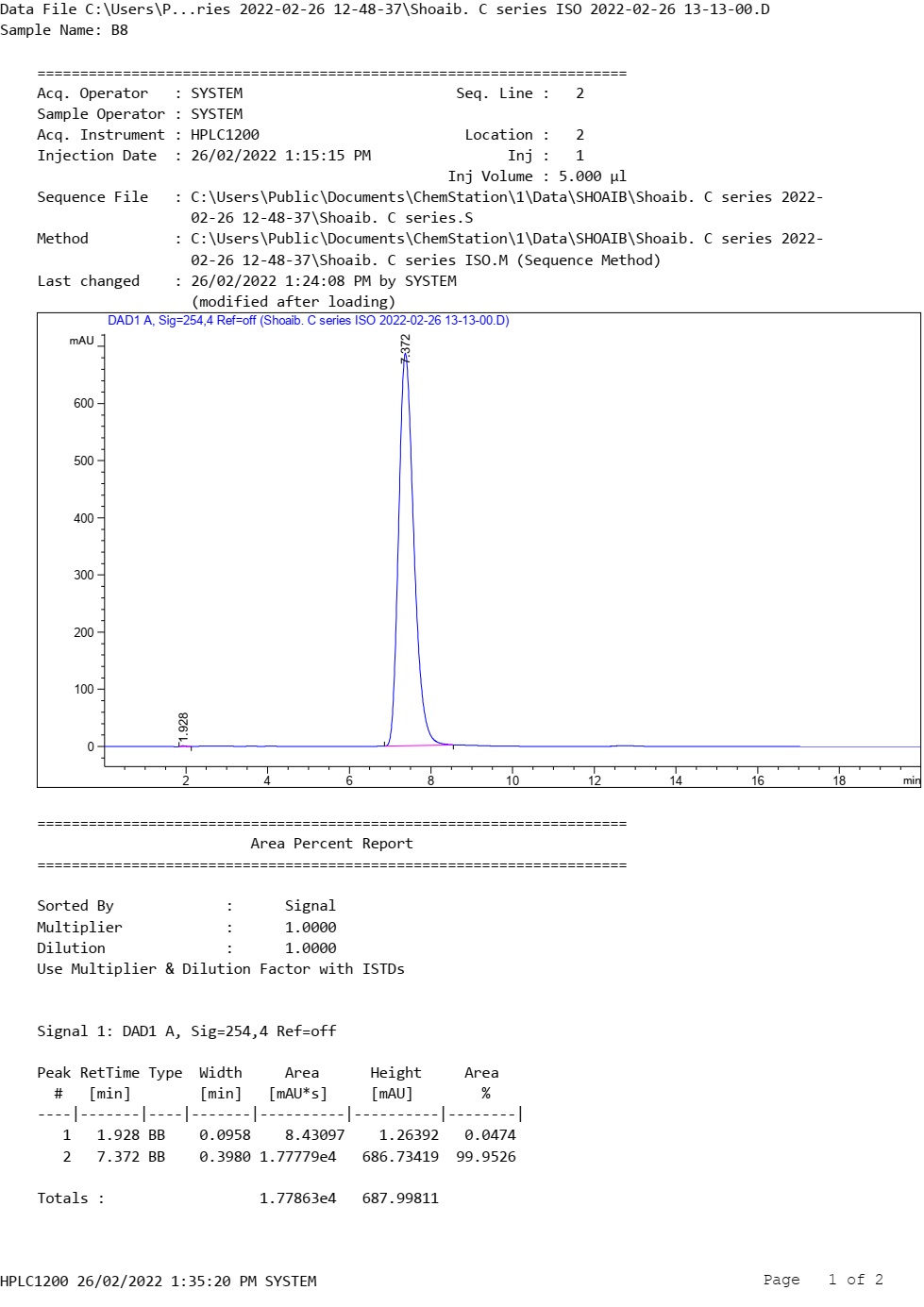


### Figure S28: HPLC Data of 10


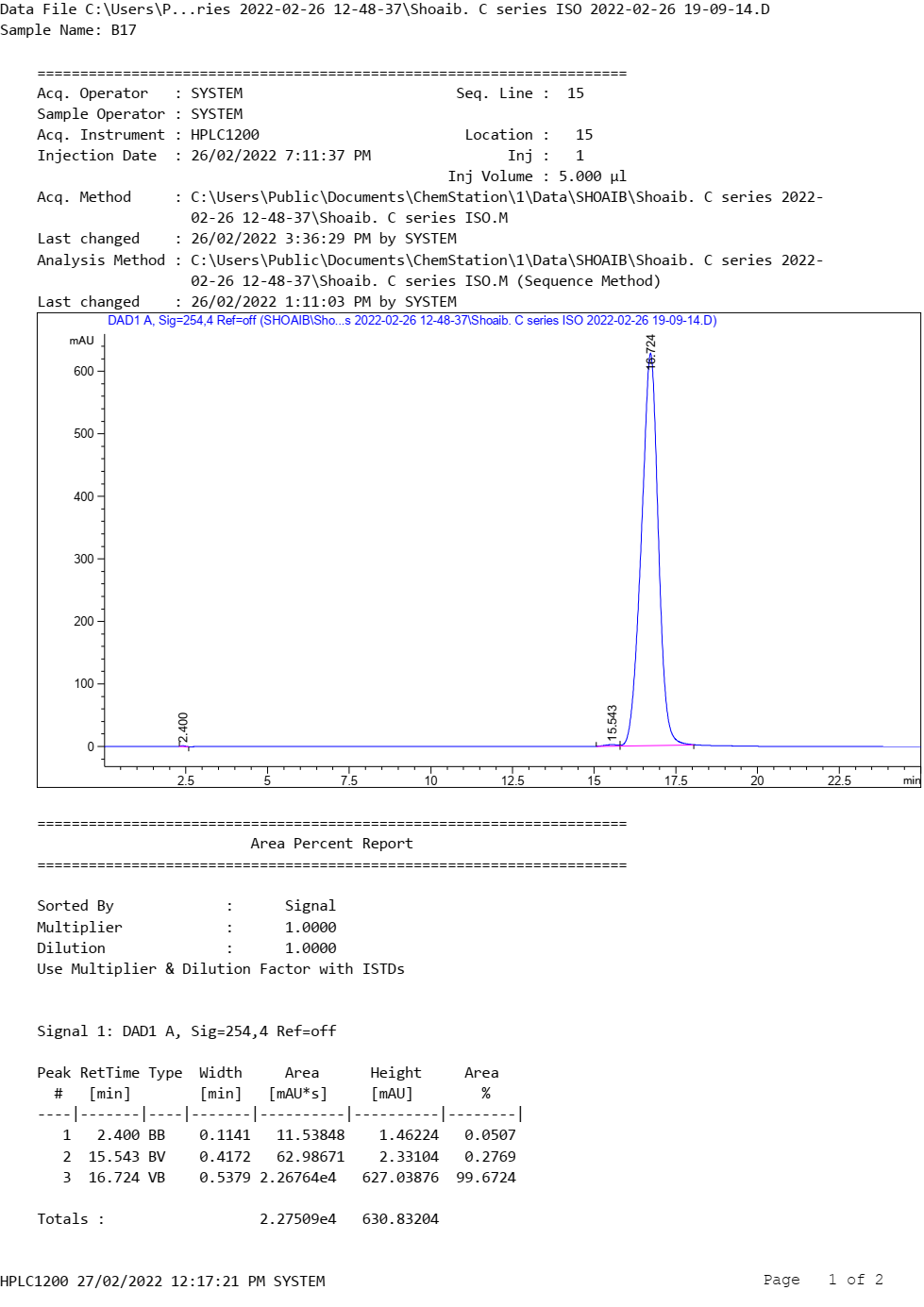


### Figure S29: HPLC Data of 11


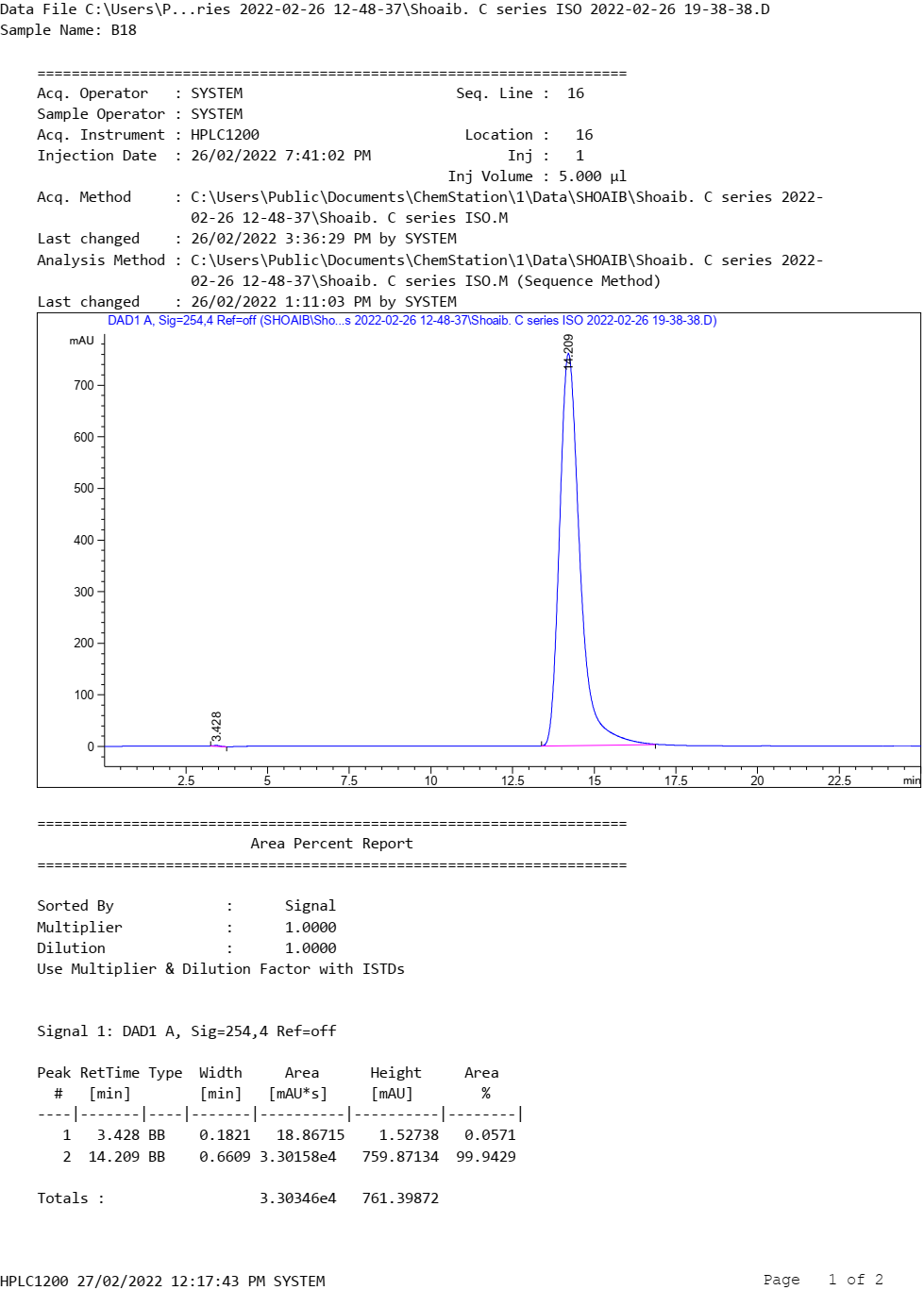


### Figure S30: HPLC Data of 12


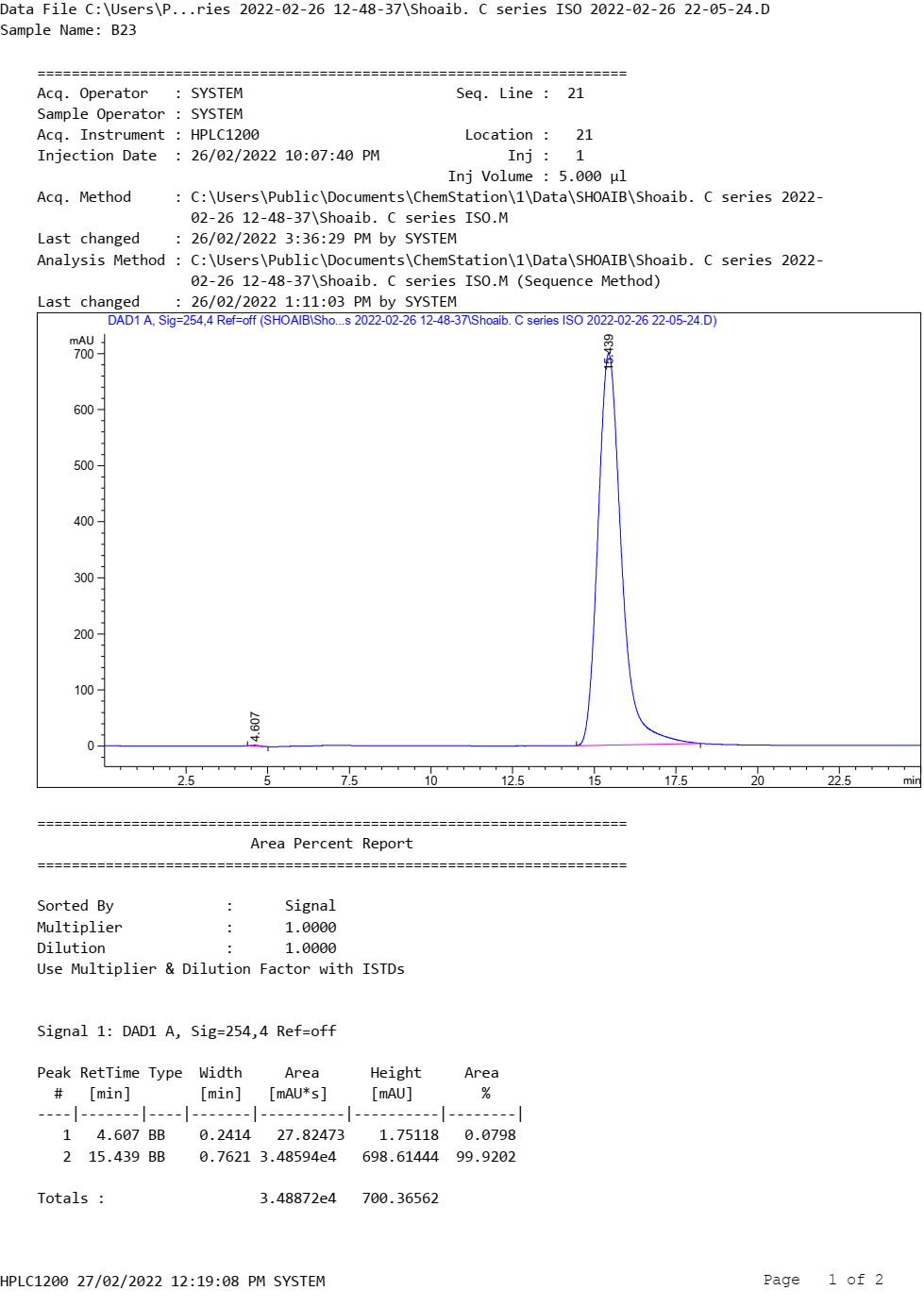


### Figure S31: HPLC Data of 13


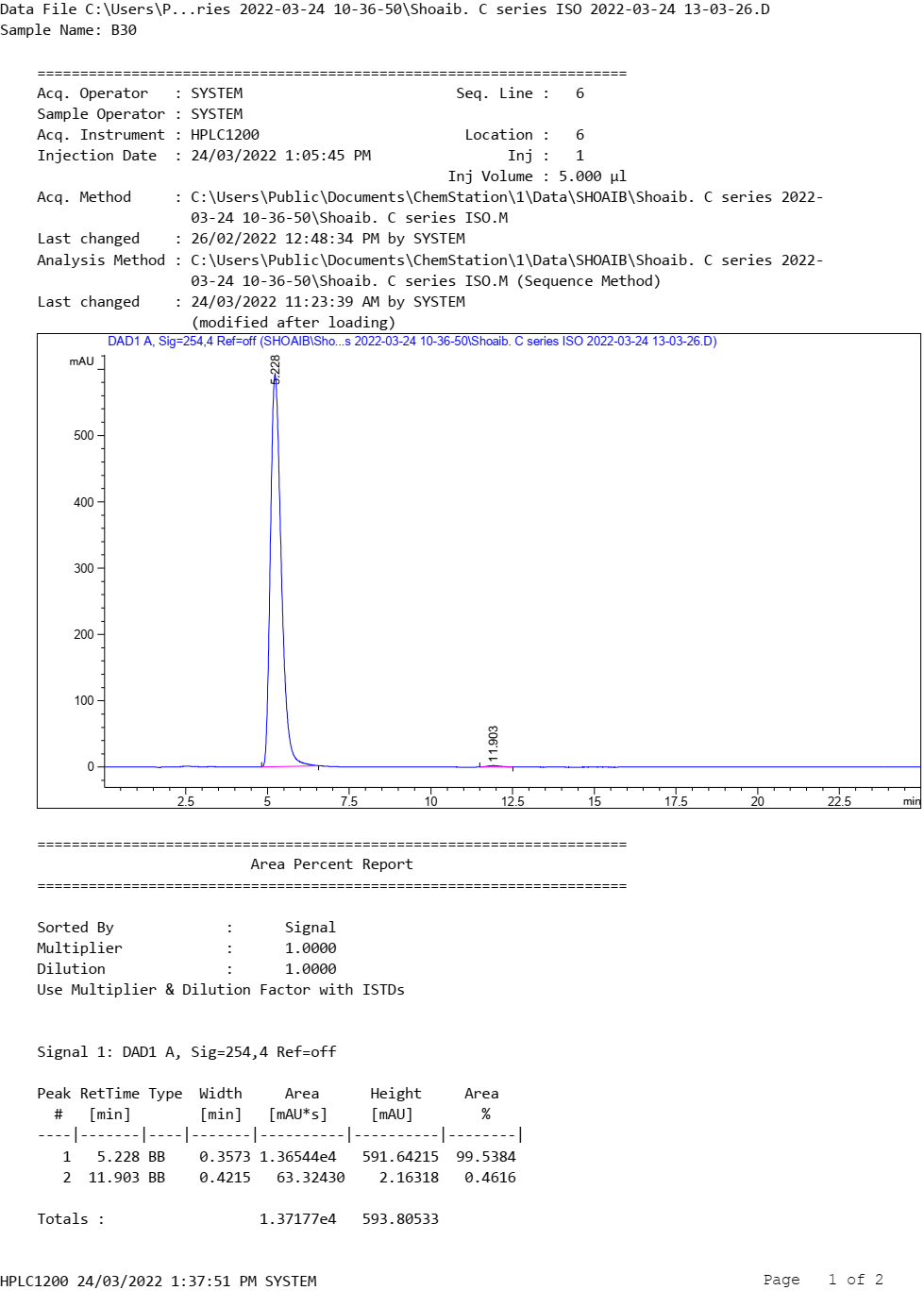


### Figure S32: HPLC Data of 14


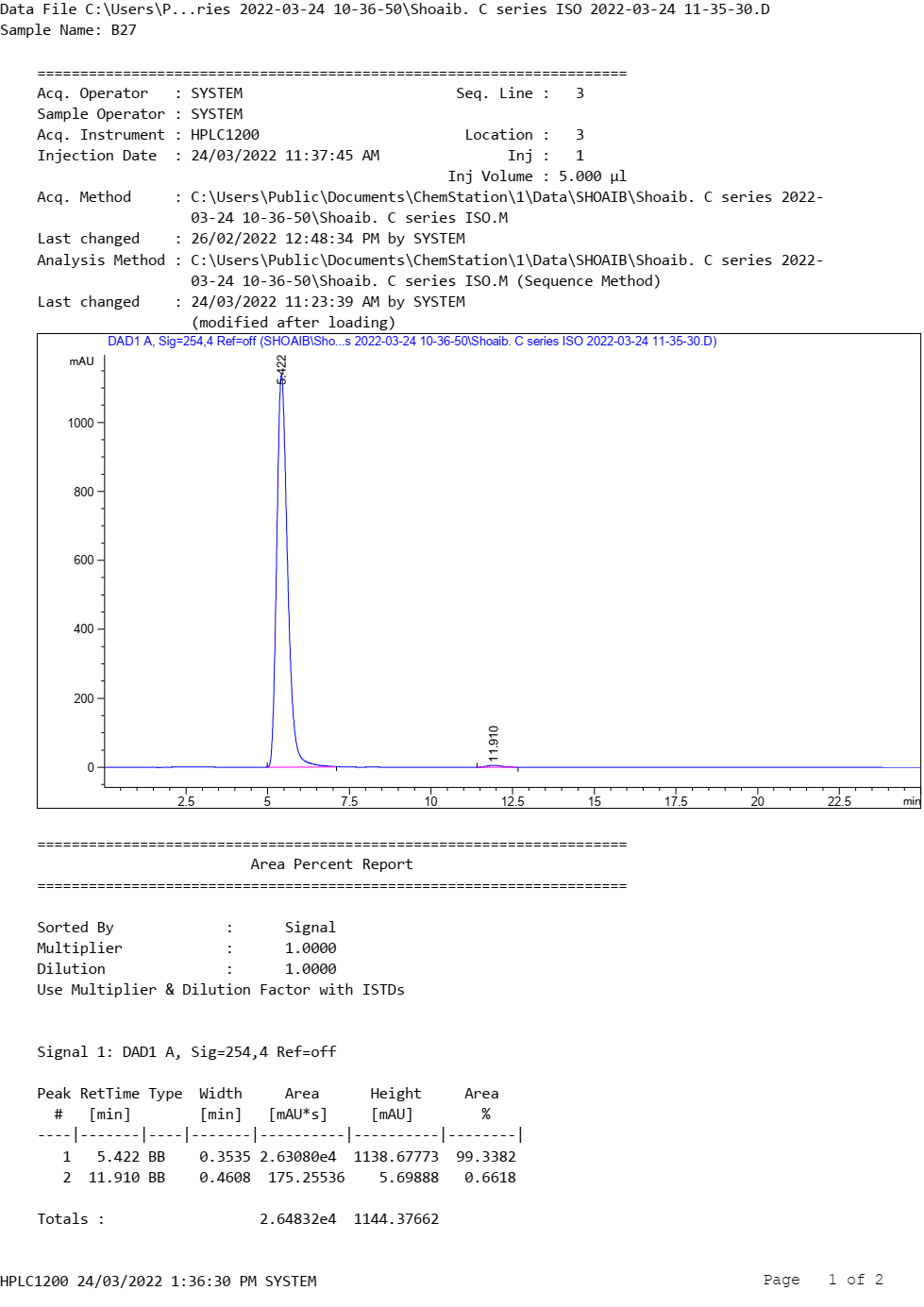


### Figure S33: HPLC Data of 15


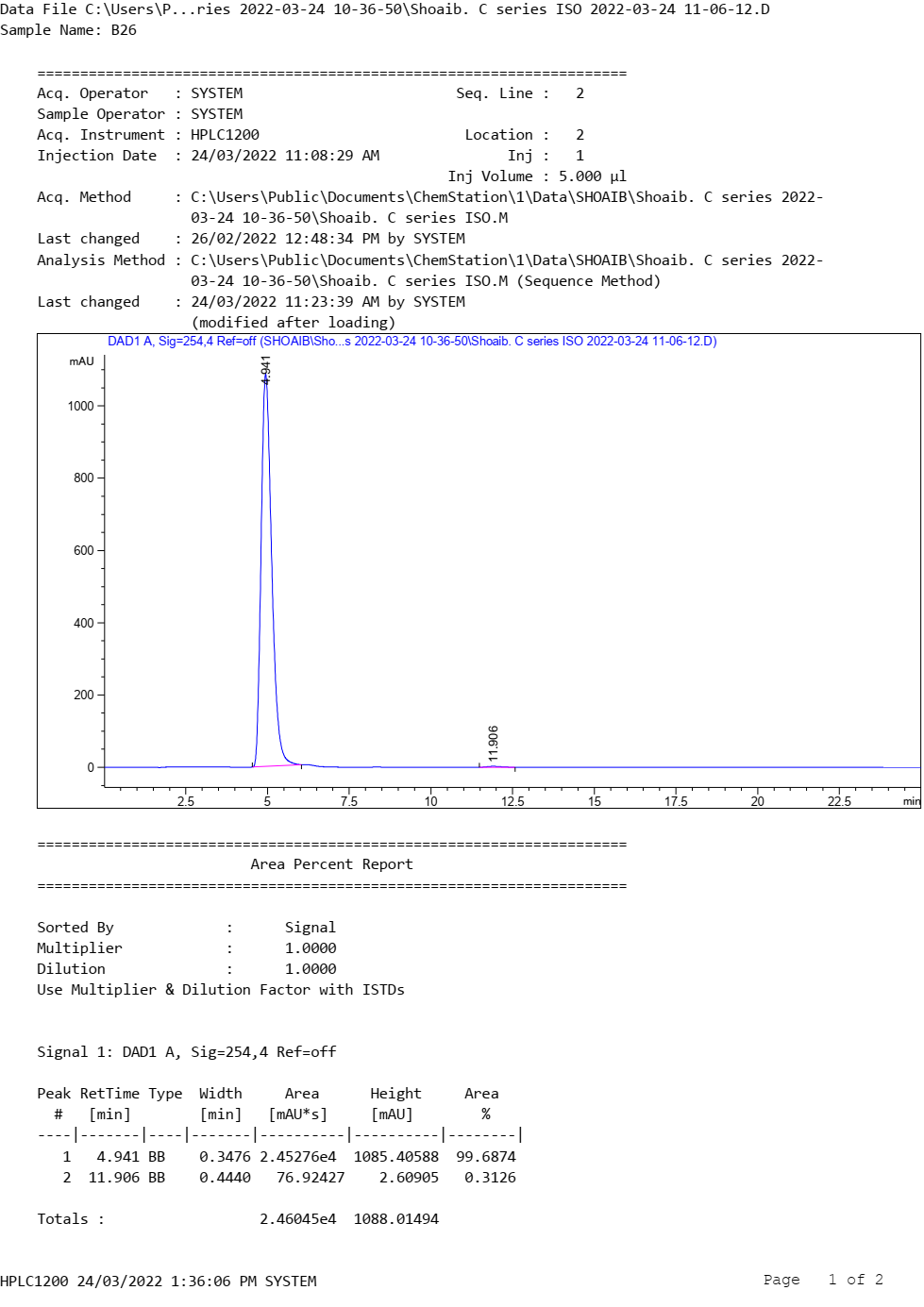


### Figure S34: HPLC Data of 16


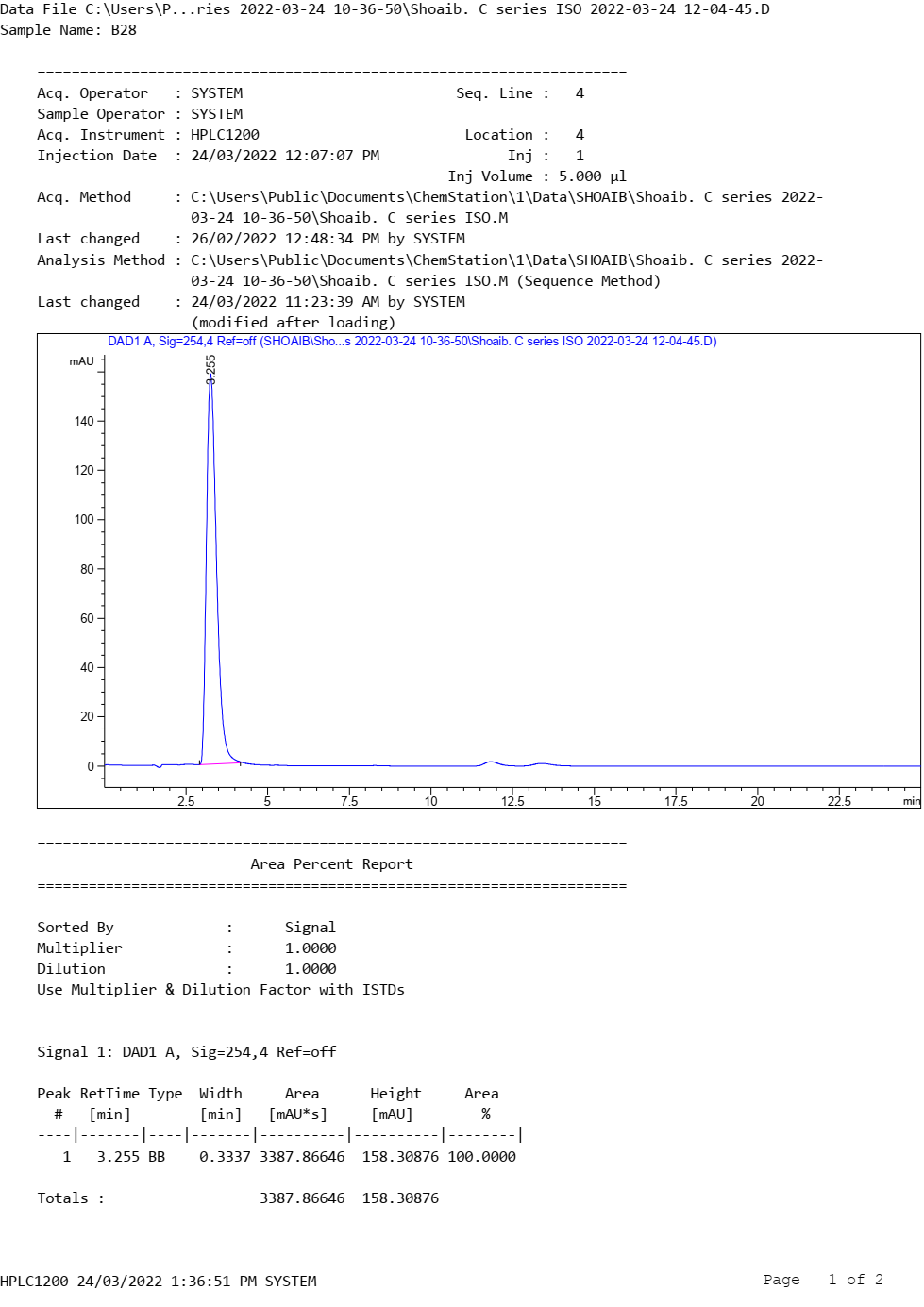


### Figure S35: HPLC Data of 17


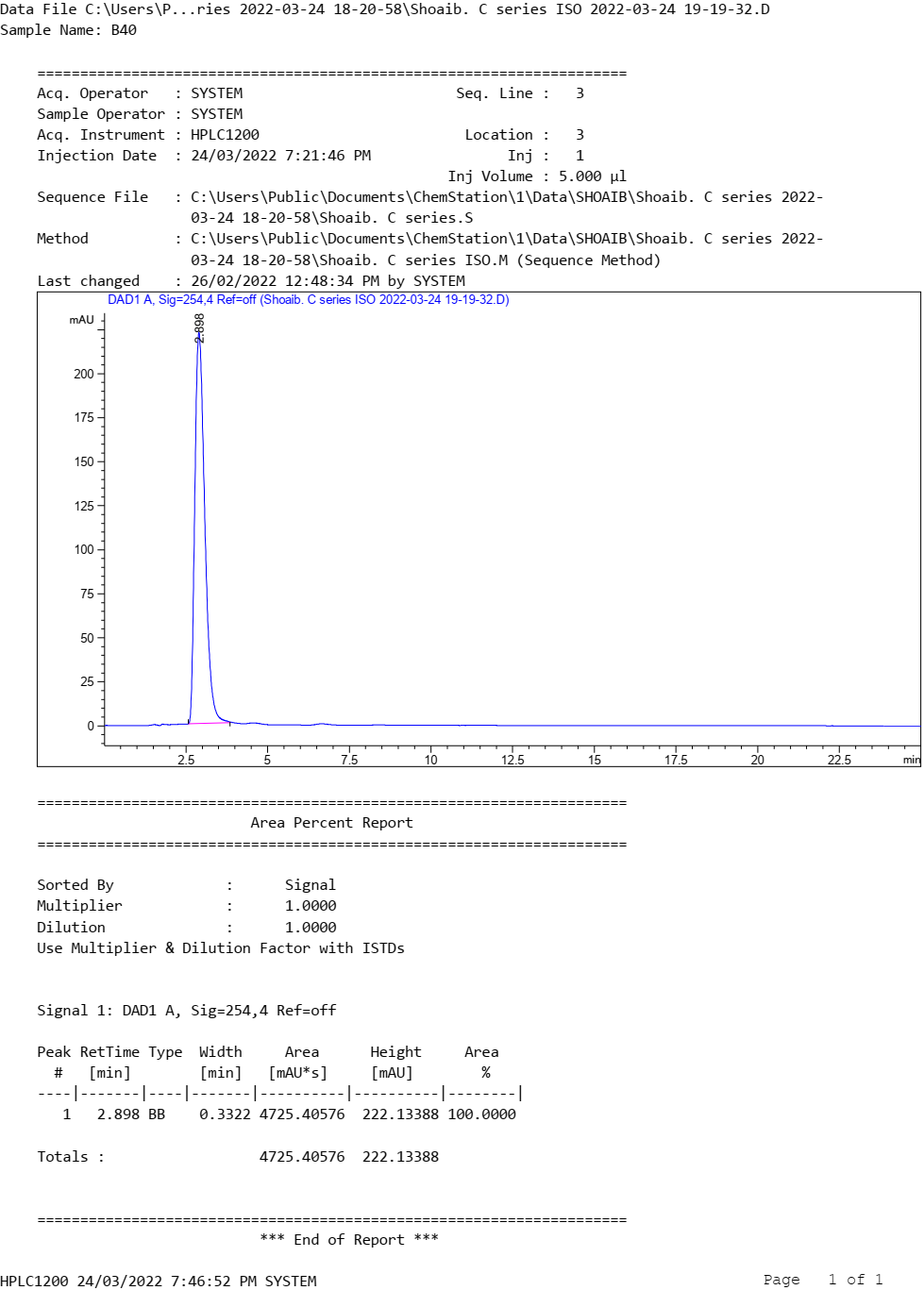


### Figure S36: HPLC Data of 18


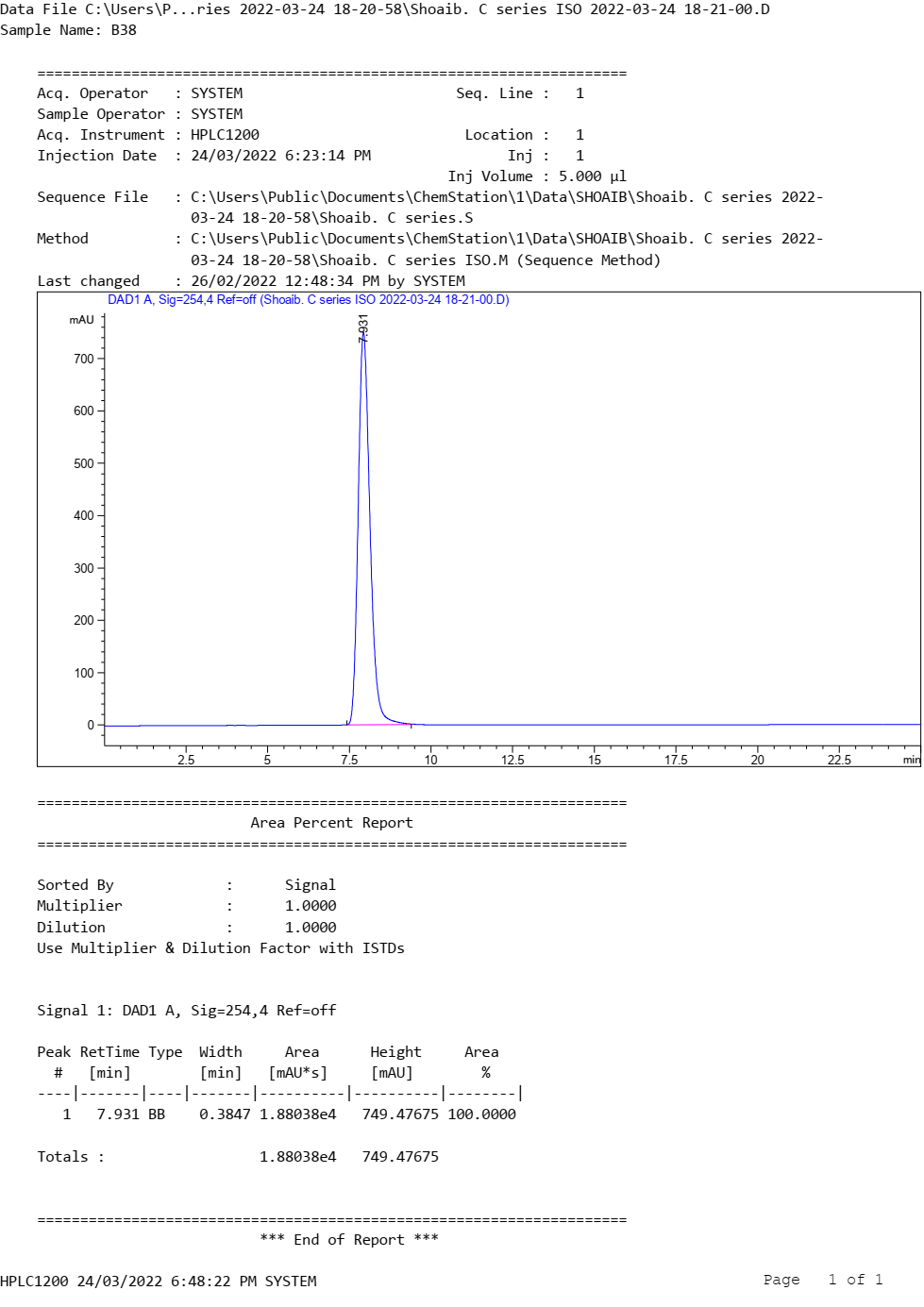


### Figure S37: HPLC Data of 19


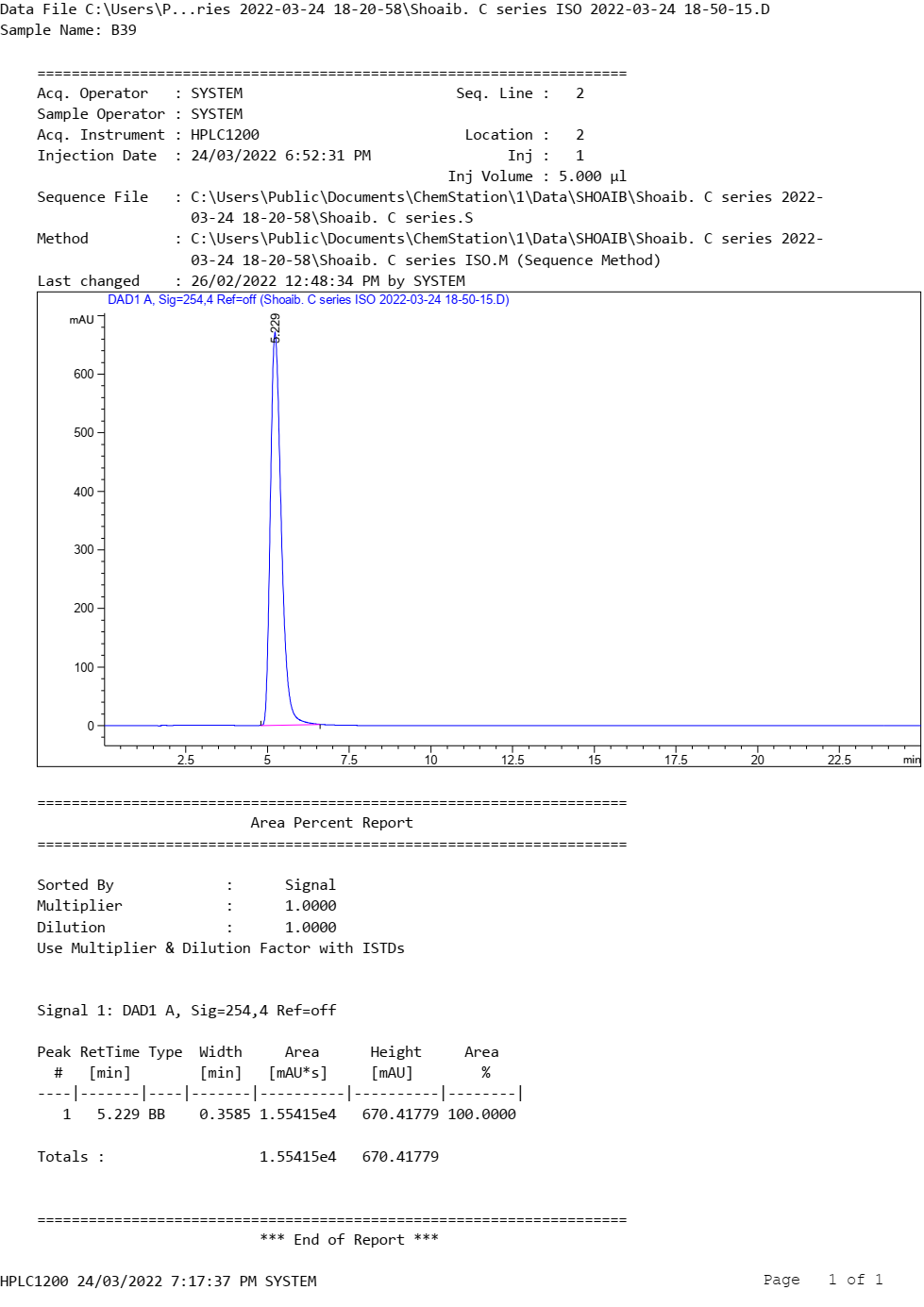


### Figure S38: HPLC Data of 20
